# Parkinson’s Disease: Bridging Gaps, Building Biomarkers, and Reimagining Clinical Translation

**DOI:** 10.3390/cells14151161

**Published:** 2025-07-28

**Authors:** Masaru Tanaka

**Affiliations:** HUN-REN-SZTE Neuroscience Research Group, Hungarian Research Network, University of Szeged, Tisza Lajos krt. 113, H-6725 Szeged, Hungary; tanaka.masaru.1@med.u-szeged.hu; Tel.: +36-62-342-847

**Keywords:** Parkinson’s disease diagnosis, Parkinson’s disease therapy, biomarkers analysis, precision-medicine methods, neurodegenerative diseases pathophysiology, translational medical research methods, communication, global health trends, clinical trials standards, artificial intelligence applications

## Abstract

Parkinson’s disease (PD), a progressive neurodegenerative disorder, imposes growing clinical and socioeconomic burdens worldwide. Despite landmark discoveries in dopamine biology and α-synuclein pathology, translating mechanistic insights into effective, personalized interventions remains elusive. Recent advances in molecular profiling, neuroimaging, and computational modeling have broadened the understanding of PD as a multifactorial systems disorder rather than a purely dopaminergic condition. However, critical gaps persist in diagnostic precision, biomarker standardization, and the translation of bench side findings into clinically meaningful therapies. This review critically examines the current landscape of PD research, identifying conceptual blind spots and methodological shortfalls across pathophysiology, clinical evaluation, trial design, and translational readiness. By synthesizing evidence from molecular neuroscience, data science, and global health, the review proposes strategic directions to recalibrate the research agenda toward precision neurology. Here I highlight the urgent need for interdisciplinary, globally inclusive, and biomarker-driven frameworks to overcome the fragmented progression of PD research. Grounded in the Accelerating Medicines Partnership-Parkinson’s Disease (AMP-PD) and the Parkinson’s Progression Markers Initiative (PPMI), this review maps shared biomarkers, open data, and patient-driven tools to faster personalized treatment. In doing so, it offers actionable insights for researchers, clinicians, and policymakers working at the intersection of biology, technology, and healthcare delivery. As the field pivots from symptomatic relief to disease modification, the road forward must be cohesive, collaborative, and rigorously translational, ensuring that laboratory discoveries systematically progress to clinical application.

## 1. Introduction

Parkinson’s disease (PD), a chronic neurodegenerative disorder, was meticulously characterized by James Parkinson in his seminal 1817 essay, “An Essay on the Shaking Palsy.” [1,2]. However, historical records demonstrate that awareness of this disease predates Parkinson’s observations, with ancient civilizations like India recognizing it as Kampavata, treated traditionally with plants such as *Mucuna pruriens*, known today as a rich natural source of levodopa [2,3]. Similarly, Greek and Roman physicians, including Galen, distinguished essential symptoms like resting tremors from action tremors, indicating an early diagnostic insight [4,5]. Furthermore, European physicians in the 17th and 18th centuries, including Hunter and Chomel, documented clinical presentations closely resembling modern PD [2,6]. Despite these earlier observations, it was Parkinson who definitively categorized the disorder by describing its hallmark motor symptoms—tremor, rigidity, bradykinesia—and proposing initial treatments such as opium and bloodletting [1,6]. His landmark essay thus established PD as a distinct neurological entity, creating a foundational understanding that catalyzed subsequent neuropathological research, notably the discovery of Lewy bodies and dopamine deficits pivotal to our current comprehension of PD [1,7]. This review begins by tracing the historical evolution of PD concepts and treatments, followed by a detailed analysis of pathophysiological mechanisms, including α-synuclein (α-syn) aggregation, genetic contributors, and environmental interactions. Subsequent sections examine the current diagnostic framework, therapeutic strategies, research gaps, and translational bottlenecks. The review concludes by proposing precision-oriented and globally inclusive research strategies to accelerate the development of disease-modifying interventions.

**Figure 1:**
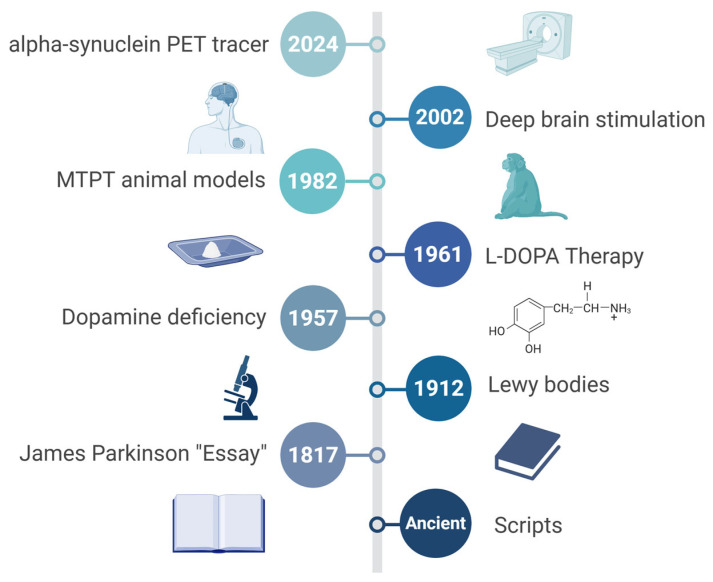
Historical Timeline of Parkinson’s Disease (PD). Key milestones chart the evolution of the field from the first descriptions of Parkinsonian symptoms in ancient medical texts, through James Parkinson’s 1817 *Essay on the Shaking Palsy*, discovery of Lewy bodies in 1912, recognition of dopamine deficiency in 1957 and the introduction of L-DOPA therapy in 1961, establishment of the MPTP primate model in 1982, U.S. FDA approves deep-brain stimulation in 2002, to cutting-edge α-synuclein PET tracers entering clinical use in 2024. Together, these pivot points illustrate how incremental breakthroughs have converged to shape today’s precision-medicine paradigm. L-DOPA, levodopa; MTPT, 1-methyl-4-phenyl-1,2,3,6-tetrahydropyridine; PET, positron emission tomography.

The neuropathological narrative of Parkinson’s, while historically anchored to post-mortem discoveries, gained profound functional clarity through modern neuroimaging and neurochemical analyses [8,9]. Initial observations by Friedrich Lewy of those enigmatic cytoplasmic inclusions became infinitely more significant with the later unmasking of a profound striatal dopamine deficit [10,11]. Have we fully appreciated, however, that this dopaminergic system degeneration is not a monolithic event? The references shared compellingly demonstrate a widespread impact, from the striatal pathways influencing motor control and cognition to the cardiac noradrenergic system, revealing a systemic deficiency that predates overt clinical signs [12,13,14,15]. This was not merely about cell death; the synaptic pathology driven by α-syn aggregation pointed to a more insidious process [16]. The consistent correlation between the burden of Lewy pathology and the loss of dopaminergic markers across various Lewy body disorders solidified a powerful, albeit challenging, central thesis [17]. A specific, critical neurotransmitter system was failing. This precise identification of a chemical culprit, a clear void in the brain’s signaling machinery, naturally and urgently paved the way for a seemingly straightforward solution: replacement. The stage was thus perfectly set for the therapeutic revolution that would follow (Figure 1).

The successful introduction of high-dose levodopa therapy in the late 1960s, a direct and brilliant consequence of identifying dopamine deficiency, truly represented a therapeutic revolution [18,19]. Spearheaded by the pioneering work of George Cotzias, this strategy dramatically alleviated the profound motor deficits of PD, seemingly overnight transforming patients who were previously rigid and immobile. This initial, almost miraculous, efficacy ushered in the “honeymoon period,” a time when it felt as though the disease had been conquered by simply replenishing the missing neurotransmitter [20]. Yet, this triumph was, in reality, a double-edged sword. Did this spectacular success inadvertently narrow our focus? The very effectiveness of levodopa reinforced a purely dopaminergic model of the disease, masking the complex, non-dopaminergic features we now grapple with [21]. Furthermore, the inevitable emergence of long-term motor complications (dyskinesias, fluctuations) shattered optimism, revealing levodopa’s symptomatic benefits without disease-modifying effects [22,23,24]. This sobering reality forced a critical re-evaluation of the disease’s nature, driving advances in delivery and resurgence in optimization strategies, including advanced formulations like intestinal gel that durably reduce ‘off’ time and improve quality of life [18,20,25,26]. Concurrently, research expanded to explore levodopa’s impact beyond motor function, including behavioral/neuropsychiatric changes in pulmonary benefits, and the ongoing debate about its potential toxicity and optimal timing [21,22,23,27].

### 1.1. Current Understanding and Clinical Definition of Parkinson’s Disease (PD)

The clinical diagnosis of PD, while seemingly anchored in the cardinal motor manifestations of bradykinesia, resting tremor, and rigidity, represents a surprisingly complex and evolving challenge [28]. For decades, diagnostic certainty has been pursued through checklists of motor signs, as exemplified by various criteria culminating in the widely adopted Movement Disorder Society (MDS) guidelines [29,30]. These criteria, while offering a structured framework and levels of diagnostic confidence, fundamentally rely on a clinical gestalt that has been progressively refined from James Parkinson’s original description to our current, more nuanced understanding [31]. However, this very reliance on a classic motor phenotype, though essential for establishing a diagnosis of Parkinsonism, increasingly appears as an oversimplification [32]. The burgeoning recognition of a vast and often debilitating spectrum of non-motor symptoms—ranging from autonomic dysfunction to cognitive and psychiatric changes—which frequently predate the onset of motor impairment, fundamentally challenges the traditional diagnostic paradigm [33]. This dissonance between our expanding knowledge of the disease’s multifaceted nature and the motor-centric criteria used for its clinical definition creates a critical juncture [34]. It forces us to question whether our current diagnostic frameworks, despite their utility, inadvertently constrain our view of the disease, especially in its earliest stages, thereby complicating the crucial task of distinguishing true PD from the wide array of other Parkinsonian syndromes [35,36].

The spectrum of Parkinsonian syndromes spans idiopathic PD, atypical tauopathies such as progressive supranuclear palsy, α-synucleinopathies like multiple system atrophy, vascular Parkinsonism, drug-induced Parkinsonism, and hereditary forms [37,38]. Clinical overlap—bradykinesia, rigidity, tremor—fuels diagnostic ambiguity, especially early in the course when therapeutic windows are widest [38]. Although validated criteria (MDS 2015) emphasize motor response to levodopa, rapid eye movement (REM)-sleep behavior disorder (RBD), hyposmia, and neuroimaging markers, sensitivity remains imperfect, and specificity declines in prodromal stages [39,40,41,42,43]. Misclassification rates still approach 20% in expert centers, undermining trial enrolment and biomarker validation [44]. Moreover, ethnicity, age at onset, and comorbid pathologies modulate phenotype, fracturing once tidy nosologies [37]. These uncertainties demand integrative algorithms that weigh longitudinal trajectories, multimodal biomarkers, and machine-learning-aided pattern recognition [39,40,45]. Clarifying nosological borders is not pedantic; it is a prerequisite for deciphering disease mechanisms. With these diagnostic fissures exposed, we now examine pathophysiological insights emerging from recent molecular, circuit, and systems-level studies that reshape our therapeutic expectations.

**Figure 2:**
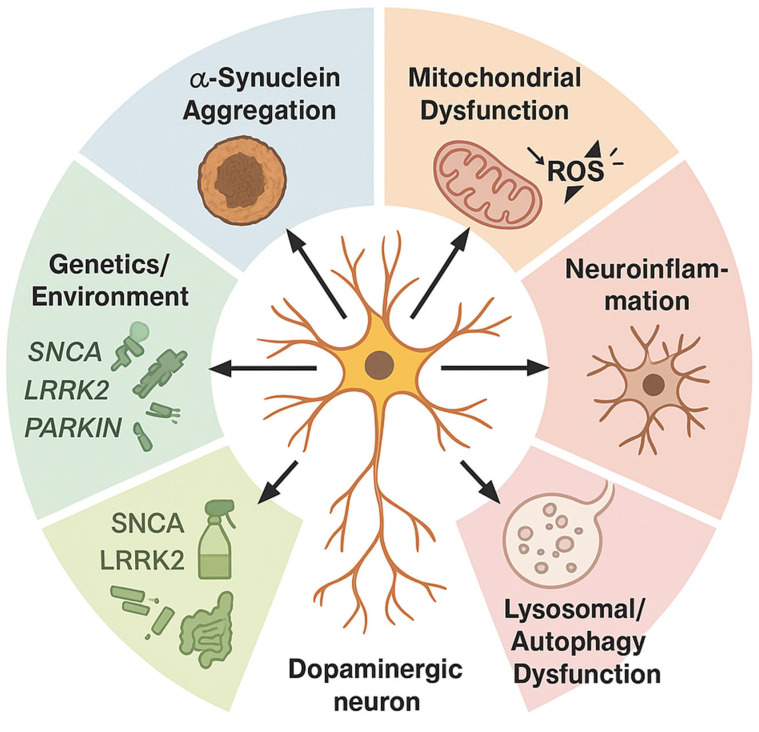
Multifactorial Pathophysiology of Parkinson’s Disease (PD). A central degenerating dopaminergic neuron receives converging insults from six interconnected pathways: (i) α-synuclein (α-syn) aggregation into Lewy bodies; (ii) mitochondrial complex-I failure with reactive oxygen species overload; (iii) chronic microglia-driven neuroinflammation; (iv) lysosomal-autophagy impairment that hampers protein/organelle clearance; (v) gut–brain axis signals, including dysbiotic microbiota and vagal transmission; and (vi) gene–environment interactions (e.g., SNCA, LRRK2, PRKN mutations plus pesticide or metal exposure). Bidirectional arrows emphasize crosstalk among mechanisms, underscoring PD as a systems disorder beyond a purely nigrostriatal dopamine deficit. LRRK2, leucine-rich repeat kinase 2; PRKN; Parkin RBR E3 ubiquitin-protein ligase; SNCA, synuclein alpha gene; ROS, reactive oxygen species.

Mechanistic studies re-frame PD as a network disorder in which misfolded α-syn seeds propagate along vulnerable connectomes, triggering mitochondrial stress, lysosomal failure, and maladaptive glial crosstalk long before dopamine neurons die [46,47,48]. In vivo positron emission tomography (PET) and cerebrospinal fluid (CSF) assays now detect soluble oligomers in prodromal RBD, linking biomarker positivity to subsequent synucleinopathy spread and cortical synaptopathy [47,49]. Experimental models further reveal bidirectional gut–brain traffic, where enteric α-syn accumulation, modulated by microbiota metabolites, primes vagal nuclei and accelerates nigral degeneration [50,51]. Conversely, suppression of synuclein alpha gene (SNCA) expression via antisense oligonucleotides or adeno-associated virus-mediated RNAi rescues motor phenotypes and normalizes striatal connectomics in rodents and non-human primates [46,52,53]. Yet, histopathological heterogeneity—co-pathology with tau, TDP-43, or cerebrovascular lesions—blurs causal inference and may underlie the mixed therapeutic responses seen in monoclonal antibody trials [54,55,56]. These converging insights ultimately recalibrate our therapeutic horizon, inviting a shift from dopamine replacement toward precision, α-syn-targeted disease modification [57] (Figure 2).

### 1.2. Overview of Therapeutic Evolution: From Levodopa to Alpha-Synuclein (α-Syn) Therapies

Levodopa has remained the unrivalled cornerstone of PD therapy since its serendipitous adoption more than five decades ago, transforming akinetic rigidity into near-normal movement within hours and thereby defining the modern therapeutic era [58,59]. Yet this simple amino-acid precursor has undergone sustained refinement: peripheral decarboxylase inhibition, catechol-O-methyltransferase (COMT) blockade, and micro-tablet or gel infusions now sculpt a pharmacokinetic profile that approximates physiologic dopamine release and mitigates pulsatile receptor stimulation [18,19,60]. Contemporary strategies advocate early, low-dose initiation coupled with sustained-release intestinal pumps or inhaled rescue formulations, while adjunct monoamine-oxidase-B and COMT inhibitors smooth residual peaks and troughs [20,61,62]. Randomized comparisons reveal that even well-titrated levodopa precipitates irreversible dyskinesia sooner than dopamine-agonist monotherapy, prompting debate over putative neurotoxicity versus disease-driven plasticity [59,63]. Parallel medicinal-chemistry pursuits have yielded antioxidant prodrugs such as SuperDopa and SuperDopamide, designed to replenish dopamine while extinguishing reactive oxygen species, thereby flirting with disease modification rather than mere symptomatic relief [64]. Despite these advances, treatment strategies remain centered on dopamine and do not yet target the upstream pathogenic mechanisms. The ensuing section dissects the inherent challenges and limitations of this dopamine-centric mindset.

Dopamine replacement transformed PD from a relentlessly disabling Parkinsonism to a treatable movement disorder, yet half a century of clinical experience has exposed its Achilles’ heel. Because levodopa is absorbed unpredictably and cleared rapidly, dopamine levels oscillate, leading to dyskinesia, motor fluctuations, and receptor maladaptation [65,66,67]. Even continuous infusion strategies only partially blunt this pharmacokinetic turbulence and cannot prevent oxidative by-products of dopamine metabolism from amplifying mitochondrial stress and glial inflammation [68,69,70,71,72]. Paradoxically, excess cytosolic dopamine may itself hasten nigrostriatal demise, challenging the dogma that “more dopamine is always better” [73]. Cognitive and limbic circuits fare no better: meta-analytic evidence shows medication-dependent gains in response inhibition erode with disease duration, underscoring a ceiling effect on non-motor symptoms [74,75]. Over time, escalating doses yield diminishing returns, spiraling polypharmacy, impulse-control disorders, and intractable freezing of gait that betray the therapy’s purely symptomatic nature [76,77,78]. Thus, despite ingenious delivery systems and receptor-selective agonists, dopamine-centric regimens remain trapped in a palliative paradigm, unable to intercept the upstream molecular cascades driving neurodegeneration [79]. This therapeutic impasse propels the field toward bold α-syn-focused platforms, gene-silencing vectors, and other disease-modifying interventions, examined next in this review.

Monoclonal and active immunotherapies that intercept extracellular α-syn now edge beyond proof-of-concept into promising territory. The anti-α-syn antibody prasinezumab slowed Movement Disorder Society–Unified Parkinson’s Disease Rating Scale (MDS-UPDRS) motor worsening for two consecutive years in the PASADENA extension, hinting at target engagement despite equivocal results in the parent trial and infusion-related events [80,81]. Parallel phase-1 vaccines, PD03A and UB-312, evoked durable, high-titer antibodies, lowered seed-competent oligomers in cerebrospinal fluid, and, crucially, demonstrated a favorable safety signal in early PD cohorts [82,83]. A shift toward multimodal modulation is also surfacing: celecoxib attenuated peripheral inflammation while reducing CSF α-syn and improving composite clinical scores in a randomized pilot study [84], whereas antioxidant co-factor ribose-cysteine preserved motor output in α-syn transgenic *Drosophila*—an intriguing blend of redox and dopaminergic support [85]. Biomarker frameworks are finally crystallizing; seed amplification assays measure disease-associated aggregates across clinical stages [86], exosomal microRNA signatures delineate molecular subtypes [87], and peripheral monocyte phenotypes mirror cognitive decline [88], enabling adaptive trial designs for rapid signal detection. Genetic insight amplifies urgency: p.A53T carriers deteriorate faster than idiopathic cases, underscoring the window for preventive intervention [89]. Together, these advances expose both the promise and fragility of α-syn-centered programs, compelling a reassessment that shapes the rationale and objectives of this review.

### 1.3. Rationale and Objectives of This Review

Despite spectacular methodological advances, PD research still drifts within critical blind spots. Proteomic screens in drug-naïve cohorts reveal promising blood signatures, yet external validation and progression tracking remain scarce [90,91]. Accelerating Medicine Partnership datasets expose demographic voids—non-white, late-stage, and early-onset cases are under-represented—hindering generalizability [92,93]. Static staging models flatten the heterogeneous trajectories unveiled by machine-learning-based progression mapping and multimodal cognitive-decline predictors [94,95]. Longitudinal connectomics demonstrate that early basal forebrain and cerebello-cortical network attrition foreshadow dementia and axial disability, but such imaging paradigms are sporadically deployed [96,97,98]. Wearable-derived digital phenotypes can identify prodromal PD years in advance, yet remain disconnected from molecular endpoints and therapeutic trials [99]. Bridging these gaps is not a peripheral academic exercise; it is the fulcrum upon which preventive, personalized, and equitable interventions must pivot. Consequently, this review interrogates unresolved questions and prioritizes strategies poised to recalibrate the PD research agenda in the years ahead.

This review surveys PD from molecular etiology to health-policy implementation, with twin aims: to chart persisting knowledge voids and to trace strategic, precision-oriented paths forward. After contextualizing historical milestones, Section 2 distils current insights into genetics, proteostasis, neuroimmune crosstalk, and environmental interplay, thereby exposing mechanistic convergence points. Section 3 and Section 4 interrogate those gaps in depth, spanning contradictory findings, methodological deficits, action–knowledge conflicts, and under-represented populations. Section 5 then converts diagnostic, biomarker, and trial shortcomings into concrete, interdisciplinary recommendations, while Section 6 synthesizes translational lessons and articulates a global, patient-centred research agenda. Throughout, critical questions, priority experiments, and policy levers are boxed for rapid reference. By threading these elements, the review furnishes researchers, clinicians, and decision-makers with a scaffold to accelerate the journey from discovery to personalized, disease-modifying care in every region.

## 2. Overview of Parkinson’s Disease (PD) Pathogenesis

PD emerges as a multifactorial neurodegenerative syndrome in which misfolded α-syn nucleates Lewy body formation, triggering microglial activation and a maladaptive unfolded-protein response. Pathogenic variants in leucine-rich repeat kinase 2 (LRRK2), glucosylceramidase beta 1 (GBA1), and Parkin RBR E3 ubiquitin-protein ligase (PRKN) intersect with pesticide-induced oxidative stress to destabilize mitochondrial quality-control networks, compromising nigrostriatal dopaminergic neurons. Resultant bioenergetic failure propagates through cortico-basal ganglia circuits, producing prodromal autonomic and cognitive derangements that precede classical bradykinesia and resting tremor. Dissecting these molecular cascades provides a mechanistic substrate for precision biomarker development and targeted, disease-modifying interventions [100].

### 2.1. Pathological Hallmarks: Alpha-Synuclein (α-Syn) Aggregation and Lewy Bodies 

#### 2.1.1. Alpha-Synuclein (α-Syn)’s Role in Parkinson’s Pathology

α-syn, a small presynaptic protein, sits at the epicentre of Parkinsonian neurobiology. Physiologically it orchestrates vesicle cycling, RNA turnover, and perhaps nuclear homeostasis, yet subtle misfolding transforms it into a toxic protein [101,102,103]. Point mutations or over-expression seed β-sheet-rich fibrils that hijack synapses, choke mitochondrial autophagy, and rally microglia into a chronic inflammatory siege [104,105,106,107]. In vivo, templated spread along connectomes converts healthy monomers into pathogenic conformers, a prion-like relay captured elegantly in rodent and primate models bearing patient-derived fibrils [108]. Despite decades of scrutiny, key riddles persist: why some neurons resist, how conformational strains dictate phenotype, and which post-translational edits gate toxicity [109]. Parsing these enigmas is not academic pedantry; it underwrites every effort to arrest disease at its molecular spark. The next step is to follow these misfolded assemblies into their macroscopic refuges—Lewy bodies—probing their formation, distribution, and pathogenic significance. Unraveling this cascade promises diagnostic biomarkers and structure-guided therapies for PD.

#### 2.1.2. Formation, Distribution, and Significance of Lewy Bodies

LBs emerge not as passive tombstones of dying neurons but as dynamic reaction vessels whose stepwise assembly—lipid scaffolding, protein sequestration, organelle capture—propels neurodegeneration more potently than fibril nucleation alone [110]. High-resolution reconstructions map concentric, multilayered cores studded with vesicles, ubiquitin, and diverse α-syn proteoforms, underscoring regulated morphogenesis rather than stochastic junk deposition [111,112]. Whether classic amyloid fibrils dominate these inclusions remains contested, yet the structural outcome appears less important than the toxic journey toward condensation [113]. Proteomic surveys of substantia nigra and patient-derived midbrain organoids converge on disrupted vesicle trafficking, mitochondrial arrest, and cytoskeletal strain as LB-associated signatures that foreshadow dopaminergic demise [114,115]. Ultrastructural diversity—somal versus neuritic, filamentous versus granular—suggests multiple biogenetic pathways, echoing “body-first” and “brain-first” propagation routes traced in post-mortem cohorts [116,117]. Age is a decisive catalyst; conditional mouse models reveal that advancing years tilt the equilibrium toward pathogenic condensation and neuron loss [118]. These spatial, temporal, and structural nuances set the stage for exploring how genetic variants and environmental exposures modulate LB biology and PD risk.

### 2.2. Genetic Contributions and Risk Factors

#### 2.2.1. Monogenic Forms: Key Genes (LRRK2, SNCA, PARKIN, PINK1)

Monogenic PD crystallizes around four pivotal loci whose mutations expose the mechanistic fault lines of neurodegeneration. Dominant variants in SNCA, which encode aggregation-prone α-syn, directly nucleate Lewy pathology and dysregulate synaptic vesicle recycling [119,120,121]. Hyper-active LRRK2 kinase alleles magnify innate-immune signaling, disturb endolysosomal traffic, and accelerate dopaminergic axon loss [120,122,123]. By contrast, recessive loss-of-function mutations in PARKIN (PRKN) and in the mitochondrial sensor-kinase PTEN-induced kinase 1 (PINK1) cripple ubiquitin-mediated mitophagy, provoking bioenergetic collapse and oxidative stress [120,124,125]. These disruptions converge: defective proteostasis, stalled organelle clearance, and chronic neuroinflammation form a triad that erodes nigrostriatal resilience and is now traceable in vivo with genetic neuroimaging biomarkers [124]. Yet genotype–phenotype correlations remain enigmatic. PARKIN and PINK1 carriers typically manifest early-onset Parkinsonism with relatively preserved cognition, whereas SNCA multiplications precipitate dementia and fulminant motor decline; LRRK2 penetrance oscillates between benign tremor-dominant phenotypes and aggressive akinetic-rigid courses across populations [123,126,127]. Consequently, therapeutic trials tailored solely to genotype have yielded clinically uneven outcomes to date [123]. Such clinical heterogeneity signals that even “single-gene” PD unfolds against a denser, still-cryptic polygenic backdrop—an arena explored next through emerging risk-profiling strategies.

#### 2.2.2. Polygenic Influences and Genetic Risk Profiling

Beyond the handful of Mendelian loci, PD reveals a polygenic architecture in which hundreds of common variants, each tiny in effect, collectively move the dial of vulnerability. Polygenic hazard scores stratify age at onset, flagging individuals who convert earlier than ancestry or sex predicts [128,129]. Network analyses map dispersed signals onto lysosomal recycling, innate immunity, and endosomal trafficking, revealing coherent biology beneath statistical noise [130,131]. Polygenic load magnifies or buffers penetrance: it propels LRRK2-G2019S or GBA1 carriers to diagnosis yet can delay symptoms when background load is low [132]. Even early-onset, ostensibly monogenic, cohorts from India demonstrate additive contributions from common variation [133]. Cross-ethnic genome-wide association studies (GWAS) now identify both shared and population-specific loci, enabling calibrated risk prediction in Asian and European groups alike [134,135]. Imaging genetics links higher scores to selective cortical thinning and subcortical atrophy, offering anatomical readouts of invisible risk [136]. Validation studies confirm discriminative capacity, yet individual prognostic certainty remains modest [137]. Yet polygenic indices ignore environmental volatility—a gap addressed in the following section, where toxins, lifestyle, and exposure histories collide dramatically (Table 1).

### 2.3. Environmental Factors and Gene–Environment Interactions

#### 2.3.1. Epidemiological Evidence of Environmental Influences

Decades of population-based studies converge on a sobering conclusion: PD clusters along environmental gradients more conspicuously than traditional familial pedigrees imply. Large-scale meta-analyses reveal that chronic exposure to organochlorine pesticides, manganese-rich welding fumes, and the solvent trichloroethylene increases risk by 60–250%, with dose–response trends persisting after socioeconomic adjustment [138,139,140]. Fine-particle air pollution (PM2.5) adds an urban dimension; cohorts exceeding 12 µg m^−3^ show elevated incidence, plausibly mediated by olfactory-bulb inflammation and systemic oxidative stress [141,142]. Even ostensibly benign lifestyle factors sculpt vulnerability: diets contaminated by organophosphates, drinking-water in agricultural basins, and long-haul occupations near highways all correlate with earlier onset [139,143]. Mechanistic triangulation links these exposures to mitochondrial inhibition, microglial priming, and epigenetic rewiring of dopaminergic nuclei, offering biological plausibility to the epidemiological signal [144,145,146]. Crucially, the attributable fraction remains mutable; policy interventions banning certain pesticides reduced regional incidence within a generation, hinting at preventability [140,147]. Such evidence mandates probing how individual genetic architectures modulate, amplify, or buffer these environmental insults—an interplay examined in greater depth in the following comprehensive section (Table 2).

#### 2.3.2. Interplay Between Environmental Triggers and Genetic Susceptibility

Mounting evidence indicates that environmental toxicants rarely act alone; their neuropathologic punch lands hardest in brains primed by inherited vulnerability. Polygenic risk scores, rare LRRK2 or GBA mutations, and common SNCA enhancers synergistically magnify the dopaminergic fallout from pesticides, heavy metals, and persistent air pollution, producing multiplicative rather than additive risk curves [148,149]. Convergent omics analyses reveal that both genomes and exposomes funnel into a limited repertoire of pathways—antigen presentation, mitochondrial quality control, and cytokine signaling—suggesting mechanistic crosstalk rather than parallel insults [142,144,150]. Epigenetic remodeling provides a pliable interface: pesticide exposure rewires DNA methylation at SNCA and immune loci, while physical inactivity or coffee consumption exert counter-modifying effects, partially buffering genetic liability [145,146,151]. Recent biobank-scale interaction screens confirm these bidirectional dynamics, yet also expose statistical fragility when ethnicity and lifestyle heterogeneity are considered [152,153]. Dissecting this molecular dialogue is pivotal, because the same immune and redox circuits that mediate susceptibility—alongside metabolic checkpoints and synaptic resilience—become tractable therapeutic targets. These intertwined threads naturally segue into a detailed exploration of neuroinflammation and oxidative stress mechanisms in PD.

### 2.4. Neuroinflammation and Oxidative Stress Mechanisms

#### 2.4.1. Neuroinflammatory Pathways in Parkinson’s Progression

Neuroinflammation is no longer an epiphenomenon in PD but a dynamic amplifier that tilts vulnerable neurons from stress to demise. Microglia, primed by α-syn and environmental toxins, shift toward a sustained M1 phenotype that floods the midbrain with TNF, IL-1β, and reactive oxygen intermediates, while reparative M2 programs collapse [154,155,156]. Concomitantly, astrocytes lose glutamate-buffering capacity and secrete complement factors that tag dendrites for removal [157]. At the signaling core, TLR2/4 and the NACHT, LRR and PYD domains-containing protein 3 (NLRP3) inflammasome orchestrate cytokine release, with GSK-3β acting as a catalytic rheostat that magnifies both pathways [158]. PET meta-analyses reveal elevated translocator protein (TSPO) binding across the striatum, cortex, and even cerebellum, underscoring the spatial reach of this immune activation [159]. Yet biomarker studies still grapple with heterogeneity: peripheral cytokine panels lack specificity, and CSF markers lag clinical progression [160,161]. Whether gut-derived lipopolysaccharide or peripheral monocytes seed the initial spark remains unresolved, complicating therapeutic timing [162,163]. Bridging these gaps demands longitudinal multimodal imaging tethered to deep immunophenotyping before oxidative stress completes the fatal cascade. Novel trials blocking NLRP3 or GSK-3β are clearly warranted now [151].

Recent work shows that microglia can amplify sterile inflammation through cGAS–STING DNA-sensing and TREM2–DAP12 signaling even when NLRP3 is genetically silenced, and pharmacological STING blockade limits dopaminergic loss in vivo [164]. Single-cell atlases of aging human midbrain reveal pronounced sexual dimorphism: female microglia preferentially engage interferon-I and cholesterol-handling programs, whereas male cells skew toward TNF–NFκB signaling, a dichotomy that correlates with faster cognitive decline in women [165,166,167]. Longitudinal epigenomic mapping has also identified a ‘resilient’ P2RY12-SALL1-positive microglial subpopulation that persists into late life and dampens α-syn seeding, highlighting endogenous brakes on chronic inflammation [168]. Together, these inflammasome-independent, sex-specific, and resilience-related mechanisms broaden the therapeutic landscape beyond NLRP3 alone.

#### 2.4.2. Oxidative Stress and Mitochondrial Dysfunction: Central Drivers in Parkinson’s Pathology

Mitochondrial redox imbalance is now considered a central driver of dopaminergic neuron degeneration [169]. Pharmacological antioxidants such as wedelolactone and melatonin restore nuclear factor erythroid 2-related factor 2 (NRF2) signaling, curb α-syn load, and rescue complex-I throughput in toxin-based models [170,171]. Vinpocetine, long known as a cerebral vasodilator, simultaneously tones down ROS, quells microglial fire, and safeguards nigral circuitry [172,173]. Non-pharmacological strategies add intriguing layers: structured exercise, hydrogen-water plus photobiomodulation, and theta-burst stimulation each recalibrate mitochondrial biogenesis or bolster glutathione stocks in early trials [174,175,176,177]. Metabolic repurposing with empagliflozin ignites the AMPK-SIRT1- peroxisome-proliferator-activated receptor-γ coactivator-1α (PGC-1α) axis, reversing rotenone lethality in rodents [178,179]. Nutraceuticals—brown-rice phenolics, anethole, and GlyNAC—extend this repertoire, hinting that diet can tweak the redox rheostat when bioavailability permits [180,181,182,183]. Yet the translational ledger remains unbalanced: sample sizes are small, endpoints diverge, and sex differences are rarely parsed. Pinpointing which oxidative nodes are actionable in humans therefore mandates a sharper methodological lens. Only through rigorously harmonized biomarkers and longitudinal phenotyping can we expose the vulnerable redox circuits (Table 3).

## 3. Core Research Gaps in Parkinson’s Disease (PD)

The forthcoming analysis delineates seven interlinked domains that continue to destabilize the evidentiary base of Parkinson’s scholarship. It opens with the variability inherent in diagnostic criteria, demonstrating how subtle shifts in inclusion thresholds propagate downstream noise. Next, it interrogates genotype–environment synergies, revealing population-specific allelic architectures that modulate toxin susceptibility and inflammatory tone. The gut–brain metabolic axis is considered thereafter, with emphasis on diet-driven microbial metabolites that skew α-syn folding kinetics. Attention then turns to experimental practice: lot-to-lot reagent drift, pre-analytical storage artefacts, and seemingly trivial changes in humidity that magnify assay variance. Statistical model selection is critiqued for its capacity to invert effect directions, while trial architecture is examined as an under-appreciated source of selection and attrition bias. The sequence concludes with a survey of multi-omic harmonization efforts, highlighting computational pipelines designed to converge transcriptomic, proteomic, and metabolomic signatures into unified pathophysiological narratives (Table 4).

### 3.1. Contradictory Findings in Parkinson’s Disease (PD) Research

#### 3.1.1. Examples of Inconsistent Studies

Human microbiome surveys paint an uneven portrait of Parkinson’s gut ecology. Several case–control studies, using 16S or metagenomic sequencing, report Prevotella depletion, Enterobacteriaceae blooms, and reduced short-chain-fatty-acid fermenters, thereby supporting a gut-origin hypothesis of prodromal disease [184,185,186]. Yet equally well-powered cohorts detect no Prevotella signal, or even observe its enrichment, once diet, constipation severity, or dopaminergic medication are rigorously controlled [187,188]. α-Syn immunohistochemistry further muddies the water: some biopsy series reveal robust colonic aggregates years before motor onset, whereas others find patchy or absent deposits despite identical sampling protocols, leaving causality in limbo [185,186,189]. Discrepancies arise from several sources. Biopsies target different gut layers and segments, so aggregated α-syn may be missed if only superficial mucosa is sampled. Antibody clones, epitope-retrieval methods, and fixation times vary across laboratories, altering detection sensitivity. Patient heterogeneity matters too: prodromal “body-first” cases often show early enteric pathology, whereas “brain-first” phenotypes may not. Finally, comorbid inflammation and constipation can mask or mimic α-syn deposits, further blurring results. Harmonized protocols and deeper submucosal sampling are therefore essential for resolving causality.

Experimental work adds more dissonance. Rotenone-exposed rats develop parallel gut dysbiosis and nigral degeneration, but the magnitude and direction of microbial shifts vary across laboratories despite standardized toxin doses [190]. Manipulating gut reactive oxygen species production rescues dopaminergic loss in some models, yet fails in others, hinting at strain-specific host factors [191]. Even Toll-like-receptor knockout mice alternately exacerbate or attenuate neuropathology depending on vivarium microflora [192]. Such inconsistencies underscore an urgent need for harmonized phenotyping, longitudinal sampling, and transparent reporting before the field can disentangle correlation from causation and pivot toward mitochondrial contradictions.

Post-mortem analyses paint a jagged picture of mitochondrial failure. Complex-I inhibition and mtDNA deletions appear near-ubiquitous in PRKN- and LRRK2-mutant mid-brains, yet idiopathic cases matched for age and treatment often show only faint respiratory drift once confounders are trimmed [193,194]. Peripheral surveys compound the puzzle: some detect systemic bioenergetic scars in muscle and skin, whereas others restrict damage to dopaminergic neurons, implying a strictly regional hit [195,196]. Even within single pedigrees, siblings may exhibit opposite mitochondrial read-outs, hinting at potent genetic modifiers or environmental buffers. Astrocytic studies add new discord—one group finds impaired oxidative phosphorylation that accelerates neuronal loss; another reports intact glial metabolism, suggesting glia could be spectators rather than culprits [197].

Pre-clinical and translational evidence is equally unsettled. Rotenone or MPTP reliably crash complex I, yet downstream phenotypes swing from fulminant cell death to reversible bradykinesia depending on strain, microbiota, and dosing schedule [198]. Suppressing PINK1-Parkin mitophagy triggers dopaminergic loss in some lines but proves benign in others, implying fallback quality-control circuits still uncharted [199]. Meanwhile, mitochondria-targeted antioxidants rescue cultured neurons, yet large trials of coenzyme Q10, creatine, and similar agents fail clinically, casting doubt on causality [200]. Inherited mitochondriopathies seldom present with Parkinsonism despite dramatic oxidative defects, amplifying the paradox [196]. Cerebrospinal and blood metabolomic panels likewise oscillate between signal and noise, stalling biomarker discovery [201]. Such contradictions demand careful unpacking—an exercise undertaken in the next section on the roots of conflicting data.

Cognitive decline presents a moving target: some longitudinal studies detect executive and visuospatial losses within two years of diagnosis, whereas others document a decade of preserved function despite similar age, education, and medication profiles [202,203]. Non-motor heralds are equally capricious. Meta-analyses portray constipation, RBD, and hyposmia as near-universal prodromes, yet prospective cohorts capture them in barely half of incident cases when symptom diaries are applied rigorously [204,205]. Even α-syn staging wavers—several autopsy series trace a neat caudo-rostral ascent, while others reveal cortical plaques that bypass the brain-stem altogether, challenging the gut-first script [206]. Blood inflammation markers add to the din: IL-6 and TNF-α predict rapid progression in clinic samples but dissolve into background noise once frailty and comorbidities are modeled in large biobanks [163].

Genetic and biomarker technologies mirror this unevenness. Polygenic scores built in European datasets falter in Asian and African ancestries, exposing hidden population-specific risk architecture [207,208]. Dopamine-transporter SPECT cleanly separates Parkinson’s from atypical Parkinsonisms at some centres but overlaps elsewhere, a gap linked to scanner harmonization and threshold choice [209]. Machine-learning pipelines boast AUCs > 0.90 for voice or gait detection in single-site studies yet drop below 0.70 when tested externally, revealing over-fitting and dataset bias [210]. Even multidisciplinary care trials produce durable quality-of-life benefits in one setting and negligible gains in another once placebo effects are stripped away [33]. The following section untangles the methodological, biological, and contextual forces that drive these conflicting data.

#### 3.1.2. Reasons Behind Conflicting Data

Several intersecting factors explain the heterogeneity across PD studies. Diagnostic variability is paramount: cohorts defined solely by cardinal motor signs differ fundamentally from those encompassing prodromal or multisystem manifestations, altering baseline biology and attenuating effect sizes of immune, genetic, and imaging markers [202,206,211]. Equally consequential is methodological divergence. Assays of α-syn-specific T-cell reactivity deploy disparate peptide libraries, detection platforms, and positivity thresholds, whereas pipelines for polygenic-risk estimation rely on distinct imputation references and ancestry weightings—differences that render inter-center comparisons problematic [207,208,212]. Population structure and environmental context add further complexity: risk alleles calibrated in European datasets underperform in Asian or African ancestries, and associations between traumatic brain injury and Parkinsonism often dissipate once granular lifestyle covariates (e.g., occupation, contact sports) are incorporated [33,207,213]. The influence of disease stage is likewise critical; immune activation or cerebellar hyper-connectivity may be adaptive in early phases yet maladaptive later, so cross-sectional studies capture divergent trajectories [202,214]. Finally, publication and survivorship biases inflate the prominence of striking positive findings—such as dramatic stem-cell graft responses—while null results are under-reported, skewing the cumulative evidence base [215].

Addressing these discrepancies demands harmonized diagnostic criteria, standardized laboratory protocols, ancestry-inclusive genetic references, and longitudinal designs capable of tracing biomarker dynamics over time. Without such methodological convergence, artefactual variability will continue to obscure genuine pathobiology and impede translational progress.

#### 3.1.3. Impact on Therapeutic and Diagnostic Development

Diagnostic innovation is advancing, yet contradictory datasets blunt its cutting edge. Candidate biomarkers—from neuropeptides to multimodal imaging signatures—show impressive accuracy in single-center studies, but performance plummets when applied to demographically distinct cohorts, complicating regulatory qualification and payer adoption [216,217,218]. Machine-learning platforms can now parse speech, gait, or PET data with area-under-the-curve values exceeding 0.90, yet external validation often exposes over-fitting and opaque decision rules; explainable artificial intelligence (AI) frameworks offer a remedy but require harmonized input standards that remain elusive [210,219]. Until diagnostic pipelines are stress-tested across ancestry, disease stage, and recording hardware, early-detection promises risk devolving into site-specific curiosities rather than broadly deployable tools.

Therapeutic development faces a parallel headwind. Divergent mechanistic read-outs—mitochondrial failure in one study, immune priming in another—have fueled a proliferation of narrowly focused interventions that shine in preclinical models but falter at phase II endpoints [220,221,222]. Network-pharmacology analyses argue that multi-target ligands or combinatorial strategies may better reflect Parkinson’s systems biology, yet such designs demand clear, consensus biomarkers for target engagement and patient stratification [223,224]. Stem-cell grafts and gene therapies illustrate both hope and hazard: proof-of-concept motor rescue is offset by inconsistent graft survival and mixed functional gains, underscoring the need for refined clinical end-points and longer follow-up [225]. In short, the field’s diagnostic and therapeutic pipelines remain tightly coupled; without harmonized, reproducible disease signatures, even the most elegant experimental treatment may never reach routine care.

### 3.2. Knowledge Voids in Pathophysiology

#### 3.2.1. Unresolved Mechanisms of Neurodegeneration

The molecular ignition of dopaminergic death in PD remains tantalizingly opaque. A growing body of work positions neuroinflammation as both spark and accelerant—microglia primed by gut-derived endotoxin, environmental toxins, or misfolded α-syn release a cocktail of IL-1β, TNF-α, and reactive oxygen species that undermines mitochondrial respiration and proteasomal flux [106,226,227]. Yet definitive triggers are still disputed: does inflammation erupt upstream of neuronal distress, or merely amplify damage already seeded by oxidative lesion in vulnerable axons? Oxidative stress itself is a moving target. Mitochondrial complex-I inefficiency, iron dysregulation, and dopamine autoxidation converge on lipid peroxidation and DNA damage, but which regulated cell-death pathway—ferroptosis, parthanatos, necroptosis—ultimately tips neurons past the point of rescue remains unresolved [201,228]. Intriguingly, invertebrate models highlight parallel modifiers seldom studied in mammals, such as lysosomal sphingolipid turnover and axonal transport genes, underscoring how single-pathway models oversimplify a web of interlocked stresses [229].

Regional selectivity compounds the puzzle. PINK1-Parkin mitophagy fails system-wide in knockout animals, yet the earliest casualties are long, highly branched nigrostriatal axons whose distal terminals accumulate calcium and metabolic debt decades before soma succumb [230,231]. Why neighboring locus-coeruleus and ventral-tegmental neurons—exposed to similar bioenergetic burden—initially survive is still debated, hinting at hidden resilience circuits embedded in calcium buffering, axonal glucose import, or astrocytic support [232,233]. α-Syn complicates matters further: oligomeric species disrupt SNARE assembly at presynaptic boutons long before inclusion bodies appear, suggesting synaptopathy precedes frank cell loss, yet whether these oligomers originate locally or arrive via prion-like spread remains contested [6,106]. Adding another layer, proteostatic failure, lysosomal acidification, and ER stress feed back into both mitochondrial dysfunction and the inflammatory cascade, creating a labyrinthine loop whose entry point varies across genetic and sporadic cases [5,8,201,231]. Disentangling cause from consequence in this self-propagating network is therefore a critical knowledge void; without it, disease-modifying therapeutics risk chasing downstream smoke rather than the original spark. The subsequent section turns to a complementary gap—the poorly mapped functions of the many genetic risk loci that modulate these intersecting pathways.

#### 3.2.2. Unknown Functions of Genetic Risk Loci

Genome-wide association studies have now catalogued more than ninety Parkinson’s risk loci, yet for the majority the causal variants, target genes, and operative cell types remain speculative at best [207,234,235]. Even celebrated exemplars reveal this opacity: only after intensive fine-mapping and CRISPR editing was GPNMB shown to bind α-syn and destabilize lysosomes, decades after the locus first emerged in linkage screens [236]. Similar detective work implicates CAMLG, a calcium-modulating adaptor at endo-lysosomal membranes, yet the precise signaling cascade by which its variant accelerates dopaminergic loss is still undefined [237]. Beyond single-nucleotide polymorphisms, common short-tandem repeats and rare coding variants contribute appreciable heritable risk, but their functional consequences on chromatin architecture or RNA splicing remain largely unexplored [238,239,240].

Functional-genomics atlases offer partial relief, uncovering enhancer–promoter loops that connect risk variants to genes governing mitochondrial bioenergetics, autophagy, and innate immunity; nevertheless, most variants sit within regulatory deserts lacking obvious molecular handles [241]. This ambiguity not only blurs hypotheses about pathogenic pathways but also hinders precision-medicine initiatives aiming to stratify patients by genetically defined mechanisms [242]. A concerted push toward single-cell multi-omics, allele-specific perturbation screens, and cross-ancestry fine-mapping is therefore imperative—topics elaborated in the following section on molecular territories still awaiting deep characterization.

#### 3.2.3. Areas Needing Deeper Molecular Characterization

Single-cell and multi-omic interrogations of Parkinsonian mid-brains have begun to lift the curtain on a molecular landscape far richer than previously imagined. Discrete dopaminergic sub-lineages, astrocytic inflammatory phenotypes, and oligodendroglial stress programs each display unique epigenomic imprints and enhancer usage, yet replication across cohorts and disease stages is still lacking [243,244,245]. Exon-focused transcriptomics reveals cryptic splicing of lysosomal and immune genes, while blood–brain correlation studies hint at peripheral windows into central pathology; however, without integrative proteomic or metabolomic layers, the biological weight of these signatures remains conjectural [246,247]. Ancestry-specific risk alleles further complicate interpretation, because many reside in regulatory “deserts” whose target genes and cell types are yet to be mapped [248].

Knowledge gaps propagate into translational pipelines. PET tracers designed to visualize α-syn fibrils, activated microglia, or mitochondrial failure perform impressively in rodent models but stumble in heterogeneous human trials, a shortfall traceable to incomplete characterization of binding epitopes and off-target effects [249,250]. Protein–protein interaction maps promise system-level insight, yet current datasets privilege abundant or easily assayable proteins, leaving low-copy but critically positioned nodes in the shadows [251]. Until chromatin architecture, metabolite flux, and interactome topology are charted with equal granularity, therapeutic targeting and biomarker discovery will continue to navigate with an incomplete compass.

### 3.3. Action–Knowledge Conflict

#### 3.3.1. Discrepancy Between Research Outcomes and Clinical Application

Efforts to refine PD into biologically meaningful subtypes have yielded elegant cluster analyses, yet the proposed taxonomies rarely inform clinic-room decisions. Many derive from highly curated trial cohorts that age more slowly and carry fewer comorbidities than community patients, so prognostic curves diverge as soon as algorithms face real-world heterogeneity [252,253]. Outcome measurement amplifies the gap: fewer than one-third of recent trials adopt the recommended core outcome set, and dysphagia or autonomic dysfunction—symptoms that often dominate quality-of-life discussions—are captured with idiosyncratic scales that defy meta-analysis [254]. Even in focused domains such as swallowing therapy, variability in endpoints thwarts synthesis and leaves practitioners guessing which protocol, if any, improves function [255].

Digital and imaging innovations tell a similar story. Machine-learning models built on single-site speech, gait, or diffusion-magnetic resonance imaging (MRI) datasets boast dazzling accuracies, yet most collapse when tested externally because preprocessing pipelines, scanner parameters, and class definitions are neither standardized nor reported in full [256,257,258]. Disease-modifying trials, meanwhile, proceed without validated biomarkers of progression, forcing reliance on crude clinical composites that lack sensitivity to short-term change; negative read-outs, therefore, reflect measurement noise as much as pharmacological failure [4,10,259,260]. Self-management and tele-rehabilitation programs fare no better—systematic reviews reveal scattered methodologies and modest effect sizes that cannot guide reimbursement or guideline development [261]. Collectively, these discrepancies illustrate an “action–knowledge” conflict: robust findings within controlled research silos falter when confronted with the complexity, diversity, and measurement inconsistencies of everyday Parkinson’s care.

#### 3.3.2. Misalignment of Preclinical Successes and Clinical Failures

Rodent toxins, transgenic flies, and even adeno-associated-virus primates routinely showcase dramatic neuroprotection—mitochondria are rescued, α-syn inclusions shrink, motor scores rebound—yet these triumphs stall once they cross the clinical threshold. A major culprit is ecological validity: most animal models capture only slivers of human pathology while sidestepping aging, polygenic load, and multisystem comorbidity, thereby overstating effect sizes and masking toxicity signals [262,263]. Trial architecture then compounds the gap. Heterogeneous patient pools, absence of molecular stratifiers, and reliance on blunt progression scales dilute true pharmacodynamic signals, so promising mechanisms die in phase II not necessarily from inefficacy but from statistical noise and endpoint insensitivity [259,264,265].

Timing adds another layer of discord. By clinical diagnosis, over half of nigrostriatal terminals have vanished, leaving scant substrate for “disease-modification”; consequently, agents that halt degeneration in toxin-treated juveniles falter in symptomatic adults [266]. Prevention strategies aimed at prodromal or genetic-risk cohorts seem better aligned, yet they hinge on biomarkers that can flag impending Parkinsonism years in advance—tools still edging through validation pipelines despite digital phenotyping and AI enhancements [267,268,269]. Even classes with robust cross-species data, such as GLP-1 and GIP receptor agonists, illustrate the gulf: variations in brain penetration, dosing frequency, and adherence have yielded equivocal human read-outs despite resounding rodent success [270]. Bridging these chasms demands next-generation models that mirror longitudinal, multisystem disease and trial designs calibrated to patient biology rather than administrative convenience, setting the stage for a critical appraisal of contemporary neuroprotective candidates.

#### 3.3.3. Examples: Neuroprotective Treatments

Neuroprotection in PD has become a crowded arena, but few entrants have advanced beyond the hopeful headline. Curcumin, selenium nanoparticles, and a widening catalogue of omega-3, lycopene, or coenzyme Q10 formulations consistently quench oxidative stress and rescue dopaminergic markers in cell and toxin-based models, yet the translation stalls at small, heterogenous pilot trials with inconsistent dosing and short follow-up windows [271,272,273,274,275]. Synbiotic regimens combining polymannuronic-acid prebiotic with *Lacticaseibacillus rhamnosus* GG extend this pattern—rodent data show amplified neuroprotection over either component alone, but human studies have yet to prove bioavailability, safety, or disease-modifying impact [276,277]. Likewise, PPAR-γ agonists, hailed for broad transcriptional control of mitochondrial and inflammatory pathways, remain trapped in phase-Ib circles awaiting a biomarker that can confirm on-target engagement in vivo [278].

Combination and advanced modalities follow similar arcs. In vitro tri-therapy with sodium phenylbutyrate, exenatide, and tauroursodeoxycholic acid yields additive rescue of neuronal viability, yet no clinical program has tackled the regulatory and pharmacokinetic complexity of three repurposed agents delivered in concert [279]. Gene and targeted-delivery platforms captivate review columns, but rigorous cost–benefit analyses and long-term safety data remain sparse [221,280]. Even mainstream symptomatic care underscores the divide: rehabilitation and surgical protocols differ widely across centers, with outcome metrics that preclude meta-analytic clarity and, thus, strong therapeutic consensus [281]. These discordant trajectories, characterized by robust preclinical mechanistic insights but limited clinical evidence, underscore an urgent need to dissect the methodological shortcomings that obstruct progress, an issue addressed in the following section.

### 3.4. Methodological Shortcomings

#### 3.4.1. Limitations in Experimental Parkinson’s Disease (PD) Models (Animal vs. Human Rele-Vance)

Classical MPTP and 6-hydroxydopamine (6-OHDA) toxin paradigms, invaluable for dissecting basal-ganglia circuitry, nonetheless model an acute, region-restricted chemical lesion rather than the slow, multisystem decline typical of idiopathic PD [282,283,284]. α-Syn over-expression or pre-formed-fibril seeding extends construct validity by reproducing Lewy pathology, yet fibril tropism and viral tropism vary widely between laboratories, yielding inconsistent timelines of synaptic failure, glial activation, and cell death [108]. Even sophisticated non-human-primate vectors replicate only fragments of prodromal dysautonomia, while failing to capture the genetic mosaicism and prolonged aging that shape human vulnerability [285]. Attempts to engineer prodromal models—sub-threshold toxins, low-dose inflammation, gut-first α-syn injections—still struggle to generate subtle non-motor phenotypes without simultaneously dampening construct fidelity [286,287]. Consequently, therapeutic candidates that rescue rapid toxin injury may address the wrong biology altogether when advanced to heterogeneous clinical cohorts.

The modeling gap widens in domains beyond nigral degeneration. Immune-dysfunction models often employ systemic lipopolysaccharide or IFN-γ dosing that eclipses physiological cytokine gradients, clouding interpretation of microglial contributions [287,288]. Computational surrogates—machine-learning classifiers built on limited, single-site datasets—exhibit dazzling accuracy in manuscript form yet fail external validation, highlighting how algorithmic “models” inherit sampling biases similar to their in-vivo counterparts [256]. No single platform, therefore, captures the intertwined choreography of aging, polygenic risk, and environmental exposures that defines real-world PD. A composite approach—cross-validating findings across cell cultures, diverse animal strains, and in silico tools—has become essential to narrow the translational gulf and to inform the rigorous biomarker pipelines discussed next.

#### 3.4.2. Biomarker Discovery and Validation Challenges

**Figure 3:**
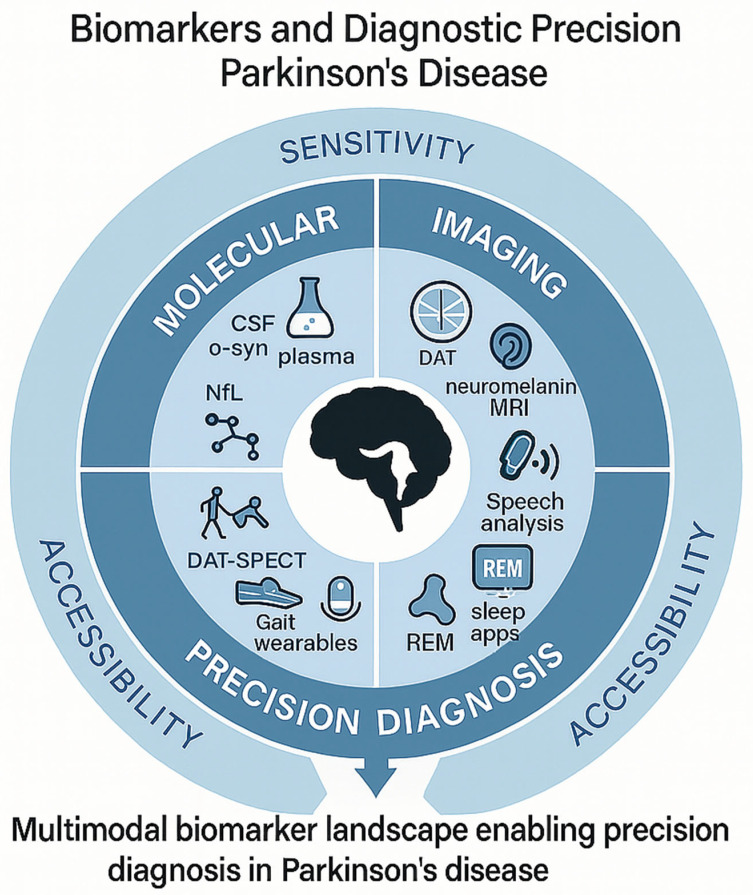
Biomarkers and Diagnostic Precision in Parkinson’s Disease (PD). Concentric rings depict three complementary domains—molecular (CSF α-syn, plasma α-syn, NfL), imaging (DAT-SPECT, neuromelanin MRI, emerging α-syn PET), and digital/clinical (wearable-derived gait metrics, speech analytics, REM-sleep apps)—arrayed around a central nigrostriatal brain silhouette. The outer band highlights key performance attributes—sensitivity, specificity, and accessibility—underscoring how integrated panels outperform single metrics and stratify patients for disease-modifying trials. α-syn, alpha-synuclein; CSF, cerebrospinal fruid; DAT, dopamine transporter; DAT-SPECT, dopamine transporter single-photon emission computed tomography; MRI, magnetic resonance imaging; NfL, neurofilament light chain PET, positron emission tomography; REM, rapid eye movement.

Efforts to identify reliable Parkinson’s biomarkers have produced an impressive shortlist—CSF α-syn seeds, plasma neurofilament light chain (NfL), dopamine-transporter imaging, metabolomic and proteomic panels—yet most fall short when confronted with heterogeneity of stage, phenotype, and assay platform. α-Syn shows excellent analytical sensitivity, but its clinical specificity erodes once dementia with Lewy bodies or multiple-system atrophy enter the comparison group, highlighting the need for composite signatures rather than single-analyte tests [216,289]. Blood biomarkers appeal for scalability, yet current candidates suffer from center-to-center coefficient-of-variation values exceeding 20%, a margin that dwarfs the biological signal in slow-progressing cohorts [290]. Metabolomic studies add breadth but rarely replicate; fewer than one-third of published panels maintain significance after independent validation, underscoring batch effects and demographic confounders [291] (Figure 3, Table 5).

CSF discovery programs, empowered by high-depth proteomics, reveal dozens of dysregulated synaptic and inflammatory proteins, but head-to-head comparisons across platforms are scarce, and longitudinal stability is largely unknown [292,293]. Neuroimaging faces parallel hurdles: PET tracers targeting neuroinflammation, mitochondrial function, or aggregated α-syn show promise in small, stage-restricted samples, yet their effect sizes diminish in mixed or early-disease cohorts, questioning universal applicability [294,295,296]. Genomic and machine-learning pipelines exacerbate the reproducibility issue—most single-nucleotide polymorphism (SNP) or multi-omic signatures identified in discovery datasets lose predictive power upon external testing, emphasizing the necessity of pooled, harmonized consortia for validation [296].

Large-scale, harmonization-driven programs now provide blueprints for reproducible biomarker science. The Accelerating Medicines Partnership–Parkinson’s Disease (AMP-PD) project releases raw genomic, transcriptomic, and clinical data under a common, cloud-based schema. The Parkinson’s Progression Markers Initiative (PPMI) aligns biosample handling and multimodal imaging across more than fifty centers, while the Michael J. Fox Foundation (MJFF) supports unified assay benchmarking and open-source analytics. Embedding future discovery and validation efforts within these infrastructures will reduce site-to-site assay drift, speed external replication, and shorten the path from exploratory signal to clinically qualified test.

Even inflammatory cytokine panels deliver conflicting results depending on assay kit and storage conditions, hampering therapeutic trial stratification [160]. Bridging these gaps will require multimodal integration, stringent replication standards, and stage-specific benchmarking—prerequisites for the longitudinal, predictive frameworks discussed in the upcoming section.

#### 3.4.3. Technological Barriers in Longitudinal and Predictive Studies

Wearable sensors, smartphone accelerometers, and Internet-of-Things (IoT) platforms promise unobtrusive capture of tremor, gait, and speech, yet most deployments are hampered by short recording windows, proprietary feature extraction, and scant attention to non-motor domains such as cognition or fatigue [297,298]. Signal drift across firmware updates, inconsistent placement of devices, and lack of calibration standards further erode longitudinal comparability, while deep-learning pipelines trained on these data rarely disclose preprocessing details, precluding replication and external validation [256]. Neuroimaging faces parallel constraints: graph-convolutional networks that predict MDS-UPDRS or Hoehn–Yahr scores from serial MRI datasets excel within single centers but falter once scanner vendors, field strengths, or motion artefacts vary [299]. The upshot is an ecosystem of clever algorithms whose performance evaporates when confronted with real-world heterogeneity.

Data fusion and interpretability add additional layers of difficulty. Multimodal machine-learning frameworks that blend clinical, imaging, electrophysiological, and biofluid variables improve prognostic accuracy, yet they demand harmonized time stamps and rigorous handling of missingness that few cohorts provide [95,300,301]. Disease-trajectory models capture individual variability and medication effects, but their complexity hampers clinical adoption and obscures causal inference [94]. Subtype-prediction algorithms trained on two well-phenotyped cohorts still misclassify up to one-third of cases when ported to external datasets, underscoring the need for broader demographic, genetic, and treatment diversity [302,303]. Until standardized pipelines, open data repositories, and transparent reporting become routine, longitudinal prediction tools will remain largely academic exercises rather than actionable instruments in routine Parkinson’s care.

Technical performance alone does not secure clinical impact. Uptake drops when devices demand frequent charging, confuse users with cryptic graphs, or ignore tremor and low-vision needs. Systematic reviews of smartwatches, insoles, and IoT systems also flag short recording windows, proprietary feature extraction, and scant coverage of non-motor domains such as fatigue or cognition. Adherence rises when dashboards convert raw sensor streams into plain-language prompts like “time for medication,” “today’s step goal achieved,” or “rest now,” and when privacy settings clearly show who can access the data. Firmware updates that break established workflows, lack of caregiver modes, and vague data-sharing policies rapidly erode trust. Simple design tweaks help: large icons and vibration cues accommodate tremor; single-button menus and wireless charging reduce daily friction. User-centered engineering can thus turn a short-lived novelty into a tool patients keep.

### 3.5. Evaluation Voids

#### 3.5.1. Absence of Standardized Evaluation Criteria for Early Detection

Efforts to flag PD years before overt motor onset still lack a universally accepted evaluative yardstick. Most prodromal studies hinge on disparate constellations of RBD, hyposmia, subtle bradykinesia, or biomarker shifts, yet thresholds for “conversion risk” differ across cohorts, leaving prevalence estimates and sample-size projections in disarray [268,304,305]. Digital sensor readouts, cognitive composites for GBA1 carriers, CSF α-syn seeds, and PET-derived synaptic density all show promise, but each is validated in isolation, on limited numbers, and with divergent statistical cut-offs [306,307,308]. Consequently, the same candidate can be deemed “high risk” under one protocol and “indeterminate” under another, hindering counseling, surveillance, and preventive-trial recruitment.

Several consensus initiatives have tried to harmonize criteria, yet none has gained the traction of the MDS clinical scale for established disease. Stakeholder workshops recommend integrating patient-prioritized functions—fatigue, speech clarity, financial decision-making—into early detection schemas, but these domains rarely align with regulator expectations for surrogate efficacy markers [306,309]. Composite indices such as PDCORE, which weave motor, non-motor, and daily-living scores into a single metric, illustrate the concept but remain tuned for manifest PD rather than prodrome [310]. Meanwhile, biomarker-directed endpoint models show how fluid or imaging signals could be mathematically linked to future disability; however, the algorithms still await external validation and cut-point standardization [308]. Until a calibrated, stage-specific framework is adopted, early-detection studies will continue to operate on shifting sands, complicating inter-study comparability and the rational staging of interventional pipelines.

#### 3.5.2. Gaps in Clinical Trial Endpoint Definitions

Regulatory setbacks in Parkinson’s drug development frequently trace back to an imprecise or ill-fitting primary endpoint. Traditional single-scale readouts—Unified Parkinson’s Disease Rating Scale (UPDRS) Part III for motor function or Montreal Cognitive Assessment (MoCA) for cognition—capture only narrow facets of an intrinsically multisystem disease and are notoriously insensitive to short-term change. Composite frameworks such as PDCORE, which blends motor scores, daily-living impact, and global impressions, offer a partial remedy yet remain calibrated to manifest disease rather than prodromal states and lack broad validation across diverse clinical settings [310]. Expert roundtables highlight parallel challenges for digital endpoints: accelerometer-derived bradykinesia indices or speech biomarkers can outperform rater assessments in controlled studies, but absent standardized acquisition protocols and statistical guidance, regulators hesitate to accept them in pivotal trials [306]. Moreover, biomarker-anchored models that mathematically couple CSF, imaging, and serum signals to functional decline are still provisional; without external replication, their predictive value remains speculative [308].

The problem sharpens in prevention or prodromal designs. Statistical simulations show that small errors in defining “conversion” from prodrome to clinical PD inflate sample-size projections by 30% or more [304,305]. Longitudinal cognitive batteries tailored to GBA1 mutation carriers illustrate how granular domain-specific endpoints can detect early decline, yet such tools are rarely embedded in mainstream interventional protocols [307]. Patient-centric frameworks that incorporate stakeholder priorities—fatigue, fine-motor dexterity, financial decision-making—have been drafted, but uptake is limited and alignment with regulatory expectations remains unclear [259,268,309,311]. In short, the endpoint landscape is fragmented, dominated by measures that neither reflect the biology targeted by emerging therapies nor resonate with lived experience. This fragmentation also explains why direct input from people with Parkinson’s, via systematic patient-reported outcomes, is still underutilized.

#### 3.5.3. Insufficient Use of Patient-Reported Outcomes

Patient-reported outcomes remain the least exploited dimension of Parkinson’s evaluation, despite mounting evidence that they capture domains invisible to clinician-rated scales. Mixed-methods interviews with individuals in early disease highlight fatigue, dream enactment, pain, and stigma as cardinal concerns, yet none of these symptoms figure prominently in regulatory endpoint menus or core outcome sets [312,313,314]. Machine-learning work combining PRO questionnaires with genotypes predicts disease severity more accurately than motor scales alone, underscoring their quantitative value [315,316]. Remote collection further broadens reach: smartphone platforms uncover sex-specific burdens of dyskinesia and sleep fragmentation that clinic visits routinely miss [316]. Even granular text analysis of verbatim posts reveals that subtle descriptions of “wobble” or “foot drag” anticipate future falls, offering low-cost surrogates for postural instability [317].

Yet methodological inertia persists. Less than one-third of recent trials incorporate any validated PRO, and those that do often ignore how comorbidities, literacy, or digital fluency reshape self-report accuracy [314,318,319]. Longitudinal data show that practice effects on cognitive tests—typically treated as nuisance variance—actually forecast long-term cognitive decline, suggesting that thoughtfully designed repeat PRO tasks could serve as early detectors of phenotypic drift [75,320]. Meanwhile, systematic reviews of digital biomarkers confirm their ability to shrink sample sizes, but without parallel patient-centric metrics they risk capturing movement physics divorced from lived experience [321]. Bridging this chasm will require co-creation of instruments with patients, rigorous validation across cultures and disease stages, and integration of PRO streams into composite endpoints so that therapeutic success is judged not only by tremor amplitude but by the voices of those who live with the disease.

### 3.6. Theory Application Gaps

#### 3.6.1. Insufficient Theoretical Integration

Despite a cascade of high-throughput omics and multimodal imaging studies, Parkinson’s research still struggles to coalesce its findings into a coherent systems-level framework. Network meta-analyses reveal wholesale re-wiring of the brain’s structural connectome—diminished segregation and weakened small-world organization—yet these macro-scale shifts are rarely modeled together with transcriptomic or proteomic perturbations observed in the very same patients [322]. Recent hierarchical attention and adaptive-sparse-learning pipelines that fuse MRI, gait kinematics, and speech dynamics improve diagnostic accuracy, but they remain proof-of-concept demonstrations housed in computer-science silos, disconnected from mechanistic biology and day-to-day clinical decision-making [323,324]. Even promising integrative network approaches that stratify longitudinal cohorts by shared genomic–proteomic signatures face data-silo barriers, limiting cross-platform reproducibility [325]. Precision-intervention roadmaps call for a move from single-node targets to dynamic, multiscale models that span molecular, cellular, and circuit hierarchies, yet concrete examples remain scarce [326].

This theoretical shortfall bleeds into management paradigms. Multidisciplinary “integrated-care” networks demonstrably enhance quality of life, but their conceptual scaffolding seldom incorporates system-biology metrics such as connectome fragility or inflammatory load, leaving clinicians to navigate by symptom check-lists rather than predictive, mechanistic dashboards [327,328]. Speech-based diagnostics, IoT wearables, and sample-dependent ensemble classifiers each promise granular phenotyping, yet they proliferate as parallel, non-interoperable pipelines [324,329]. The result is a patchwork of unlinked biomarkers and intervention strategies that obscure emergent properties of the disease network. Such fragmentation invites a reflexive return to single-pathway, reductionist models—approaches explored in the following discussion of how over-reliance on simplified experimental systems continues to constrain progress.

#### 3.6.2. Over-Reliance on Reductionist Models

Classical toxin or over-expression paradigms remain the workhorses of pre-clinical discovery, yet their reductionist focus on rapid nigral loss obscures the multisystem, decades-long evolution of idiopathic PD. Acute MPTP lesions provide clean motor phenotypes but ignore prodromal dysautonomia, mood change, and sleep disruption; even sophisticated α-syn fibril or viral models show laboratory-dependent variability and seldom recapitulate circadian-rhythm instability or mixed proteinopathies that typify advanced PD [282,284,285,330,331]. Attempts to diversify model systems—zebrafish for high-throughput screens, drosophila for genetic interaction maps—add breadth yet still fragment the disease into isolated pathways, leaving unanswered how dopaminergic stress interlocks with systemic immunity or metabolic aging [331]. Consequently, candidate therapeutics that rescue a single lesion cascade often falter when confronted with the layered pathobiology of human trials.

A similar narrowing pervades data-science pipelines. Voice-only classifiers, dimensionality-reduction on gait kinematics, or light-gradient-boosted models that hinge on one dominant feature—bradykinesia, hyposmia, a risk SNP—deliver striking accuracy within curated cohorts but shed performance once heterogeneous, medication-modulated populations enter the frame [94,332,333,334]. Statistical progression models that accommodate overlapping trajectories offer a corrective, yet without biological annotation they risk becoming elegant curve-fitting exercises divorced from mechanism [8,94]. Multimodal fusion frameworks exist but are often siloed within computational groups and rarely validated alongside wet-lab findings, perpetuating a piecemeal view of PD pathogenesis. Overcoming these blind spots will demand sustained, cross-disciplinary dialogue that links bench biologists, modelers, and clinicians around shared, integrative hypotheses.

#### 3.6.3. Limited Cross-Disciplinary Collaboration

Cross-disciplinary work in PD has advanced from isolated pilot projects to nascent regional and global networks, yet it remains hampered by structural, cultural, and financial barriers. German baseline audits show that neurologists, physiotherapists, nurses, and social workers still document in siloed electronic systems; reimbursement models reward single-specialty encounters rather than joint case reviews, making sustained cooperation difficult [335,336,337]. Even when multidisciplinary meetings occur, pharmacists, speech therapists, and dentists—professionals who can mitigate polypharmacy errors, dysphagia, or oral infections—are seldom invited, despite clear patient demand [338,339]. Qualitative interviews across Europe, Asia, and North America echo this fragmentation: people with PD and their carers describe disjointed hand-offs, duplicated testing, and conflicting advice that erode confidence in the healthcare journey [340,341]. Multidisciplinary team models do improve medication optimization and quality-of-life metrics, but detailed blueprints for governance, shared metrics, and digital communication tools remain poorly disseminated, hindering scale-up beyond early adopters [342].

Research networks provide a contrasting, if uneven, benchmark. The Global Parkinson’s Genetics Program (GP2) and the MJFF Global Genetic PD Project have demonstrated that harmonized consent templates, cloud analytics, and regional capacity-building grants can unite more than a hundred institutions across income settings, accelerating variant discovery while amplifying under-represented voices [343,344]. Yet such collaborative energy has not permeated clinical-implementation science; longitudinal cohorts and interventional trials still recruit predominantly from North American and Western European tertiary centers. The resulting data blind spots make it difficult to generalize findings or tailor interventions for diverse populations. Addressing these gaps demands frameworks that integrate basic scientists, data engineers, allied-health professionals, and community stakeholders around shared hypotheses and equitable resource allocation—concerns that become even more pressing when the field turns its attention to systematically underrepresented cohorts.

### 3.7. Underrepresented Cohorts

#### 3.7.1. Lack of Diversity in Genetic Studies

Most genome-wide association studies that underpin today’s catalogue of ≈ 90 common PD risk loci were assembled almost exclusively from individuals of northern-European ancestry; analyses suggest that fewer than 15% of all genotyped cases originate from Africa, Latin America, or South Asia [207,248,345]. Linkage disequilibrium patterns, haplotype architectures, and allele-frequency distributions differ markedly across populations, so European-trained polygenic scores lose predictive power elsewhere and ancestry-specific variants of large effect remain invisible. Illustrative gaps have begun to surface. A recent GWAS of >7000 African and African-admixed participants revealed a novel intronic GBA1 signal that modulates PD risk independently of the well-known p.N370S allele—an association that would have been missed in European cohorts [346]. Conversely, targeted sequencing in mainland China confirmed PRKN as the most frequent Mendelian contributor and showed that a molecular diagnosis can anticipate motor onset by more than ten years, underscoring the need for region-tailored panels [347].

Momentum toward broader inclusion is growing but scale remains modest. Latino investigations show a mosaic of Indigenous, European, and African haplotypes that reshuffle fine-mapping priorities and compel admixture-aware analytics [348]. New programs—GP2 and the MJFF Global Genetic PD Project—provide cloud pipelines, harmonized consent, and training grants to sites in Africa, South America, and Southeast Asia, yet their enrolment targets are still far from closing the representation gap [349,350]. Multi-omic resources capturing cell-type diversity across ancestries are rarer still, limiting systems-biology models that might explain why identical mutations exhibit variable penetrance in different genetic backgrounds [351]. Until diversity is treated as a scientific prerequisite rather than an aspirational add-on, precision-medicine promises will remain unfulfilled—a reality that becomes even starker when one examines who is actually recruited into interventional studies, the subject of the next discussion on underrepresentation in clinical trials (Table 6).

#### 3.7.2. Underrepresentation in Clinical Trials

Despite repeated calls for inclusivity, demographic audits reveal Parkinson’s trials still enrol a narrow slice of the population. Meta-analyses show fewer than one in five studies even report participant race or ethnicity, and when they do, Black and Hispanic patients together seldom exceed 5% of the sample [352,353,354]. Catechol-O-methyl-transferase inhibitor trials, for example, drew almost exclusively from White European cohorts, despite differential COMT allele frequencies that could modulate efficacy and safety in other ancestries [352,353,354]. Barriers span mistrust, travel burden, restrictive eligibility, and recruitment pipelines that favor tertiary centers located far from minority or rural communities [355,356]. Age bias is equally pervasive: 92% of recent randomized trials either explicitly cap enrolment below 80 years or deploy exclusion criteria (polypharmacy, cognitive screening cut-offs) that effectively sideline the oldest—and most clinically relevant—patients [357]. Sex representation fares only marginally better; women constitute barely 30% of infusion-therapy trial enrolees, even though pharmacokinetics, dyskinesia risk, and caregiving burdens differ by sex [358].

Interventions to correct these skews remain piecemeal. Community-engagement frameworks such as Fostering Inclusivity in Research Engagement for Underrepresented Populations in Par-kinson’s Disease (FIRE-UP PD) demonstrate that partnering with faith organizations, deploying mobile research units, and compensating travel can double minority recruitment, but such strategies have yet to be adopted at scale [359]. Guideline proposals advocate mandated demographic reporting and enrolment targets tied to disease prevalence, yet few sponsors incorporate these metrics into study milestones or funding contingencies [353,355,360]. Until trial designs are re-engineered to accommodate linguistic diversity, caregiver participation, and comorbid conditions common in older adults, the evidence base guiding new therapeutics will continue to under-represent those most likely to use them—an imbalance with direct consequences for efficacy, safety, and real-world generalizability.

#### 3.7.3. Consequences of Biases for Therapeutic Efficacy and Generalizability

Therapeutic evidence generated in demographically narrow samples rarely travels well. Trials that exclude adults over 80—a practice affecting 92% of recent PD RCTs—produce dosing guidance that fails older patients, whose poly-pharmacy profiles, frailty indices, and device-handling capacities differ materially from those of younger cohorts [357]. Similarly, gait-targeted interventions engineered from meta-analytic averages of European trial volunteers overlook cadence and stride-length norms that vary by ancestry and cultural walking habits, limiting external validity when rolled out in more diverse clinics [361,362]. Biases also infiltrate real-world implementation: access to device-aided therapies hinges on clinician referral patterns and patient self-advocacy, both of which are skewed by socioeconomic status, language fluency, and implicit expectations of adherence [363]. The end result is a pipeline in which promising technologies stall at the point of delivery, widening health-equity gaps rather than closing them.

Under-sampling of women, minorities, and the very old also warps mechanistic science. Pharmacogenomic modifiers of levodopa- and dopamine-agonist metabolism differ by sex and ancestry; yet dosing algorithms seldom incorporate these variants because discovery cohorts are too homogeneous to detect them [364]. Self-management trials that do succeed in recruiting diverse participants reveal heterogeneity in digital literacy and comorbidity burdens, producing effect sizes that diverge sharply from earlier, less inclusive studies [261,365]. Even “objective” AI classifiers inherit their training biases: narrative reviews show that speech and handwriting models validated on limited dialects or alphabets misclassify minority users, undermining early-detection ambitions [366]. Herbal, stem-cell, and non-pharmacological meta-analyses read similarly—promising signals appear, but sample sizes are small and demographic reporting sparse, leaving dose–response relations and safety profiles uncertain in underrepresented groups [367,368,369]. Until inclusivity becomes a non-negotiable criterion—from recruitment targets to algorithm training sets—efficacy claims will remain conditional, and the dream of generalisable, precision Parkinson therapy will remain out of reach.

## 4. Beyond Alpha-Synuclein (α-Syn): Emerging Therapeutic Targets and Approaches

The forthcoming subsection shifts the lens from protein-centric dogma to a systems-level rescue plan. It first details how restoring lysosomal flux, rebalancing mitochondrial fission–fusion cycles, and tempering neuroinflammation can unblock dopaminergic survival pathways. The narrative then pivots to precision neurology, where multi-omic fingerprints and real-time phenotyping calibrate therapy to each patient’s molecular liabilities rather than to a statistical mean. Finally, it shows that small-molecule cocktails, allele-specific gene editing, and rigorously tailored lifestyle regimens can be woven into synergistic, adaptive care. Together, these themes announce a decisive move toward bespoke, multimodal disease modification.

### 4.1. Novel Targets: Lysosomal Pathways, Mitochondrial Dynamics, Neuroinflammation Modulation

Disruption of the autophagy–lysosomal axis has emerged as a central bottleneck in dopaminergic neuron survival, making it an enticing therapeutic command post. Small-molecule chaperones that stabilize mutant β-glucocerebrosidase (GBA) are already able to decongest lysosomal traffic jams and curb α-syn accumulation in pre-clinical models, while LRRK2 kinase inhibitors and Parkin activators synergistically restore endo-lysosomal sorting and mitophagy, respectively [222,370] These interventions illustrate a strategic pivot: rather than merely sweeping up misfolded proteins, we can re-engineer the cell’s recycling plant and mitochondrial conveyor belt in tandem. The same reasoning underlies newer mitochondrial “dynamizers” that fine-tune fission–fusion cycles and rescue bioenergetic failure—an approach recently extended to Abelson and Rabphilin-3A signaling nodes, which orchestrate vesicular turnover at the synapse [371]. Collectively, these pipelines sketch a future in which PD is tackled as a disorder of intracellular logistics, not simply proteinopathy.

Yet neurons do not degenerate in isolation; they succumb within a storm of glial activation, peripheral immune crosstalk, and gut–brain mis-signaling. Modulating neuroinflammation has therefore vaulted onto the shortlist of disease-modifying strategies. Colony-stimulating factor-1 receptor blockers temper microglial proliferation, while NLRP3 inflammasome inhibitors—and, intriguingly, gut-targeted microbiome correctives—dampen the chronically primed innate response that accelerates synaptic demise [371,372,373]. Parallel efforts to boost endogenous dopamine by stabilizing tyrosine hydroxylase phosphorylation hint that metabolic rewiring can be layered atop immunomodulation to widen the therapeutic aperture [374]. The constellation of candidates now spans gene therapy vectors, immunotherapies, small molecules, and engineered peptides—diverse tools converging on a shared goal: to recalibrate the intracellular and intercellular ecologies that dictate neuronal fate [220,221,375]. Still, heterogeneity in genetic architecture, prodromal trajectories, and inflammatory tone means that no single target is likely to suffice. Bridging these mechanistic insights with multi-omic biomarkers will therefore be crucial, setting the stage for the next frontier—personalized medicine and precision neurology potentials—where intervention is matched to the individual’s molecular fingerprint rather than to a one-size-fits-all disease label.

### 4.2. Personalized Medicine and Precision Neurology Potentials

Precision neurology in PD is moving beyond population-level algorithms toward the granular resolution of each patient’s genomic, proteomic, and exposomic fingerprint. Pathogenic variants in GBA, LRRK2, and PRKN, once viewed merely as risk markers, now dictate enrolment in allele-matched trials of chaperone therapy, kinase inhibition, or mitophagy enhancement—an approach that has already revealed stark response heterogeneity between mutation carriers and idiopathic cases [221,370]. High-throughput single-cell transcriptomics and machine-learning-derived digital phenotyping further subdivide “idiopathic” PD into transcriptomic endotypes characterized by distinct signatures of mitochondrial insufficiency, lysosomal stress, or neuroimmune priming [376,377,378]. These sub-clusters serve a dual purpose: they refine prognostication and, more importantly, enable adaptive trial designs in which drug allocation is tethered to a patient’s dominant molecular liability rather than to a one-size-fits-all disease label [379]. By embedding such stratifiers into platform studies we can accelerate the go/no-go decision-making that has historically bogged down disease-modifying pipelines.

Personalized medicine cannot, however, ignore the dynamic crosstalk between neurons, glia, and peripheral organs. Patients with a neuroinflammatory or gut-microbiome-driven endotype, for example, appear most likely to benefit from CSF1-receptor antagonism, NLRP3 inhibition, or microbiota re-engineering, whereas those with synaptic vesicle trafficking deficits may respond preferentially to emerging Protein Abelson or Rabphilin-3A modulators [220,371]. Stem-cell-derived dopaminergic progenitors and in vivo gene-editing vectors offer another layer of personalization, supplying bespoke cellular “replacement parts” or allele-specific repair kits that bypass the pharmacodynamic ceiling inherent to small molecules [225,380]. Yet even the most precisely targeted intervention risks futility if deployed in isolation, given the multifactorial nature of PD pathogenesis and progression. Precision neurology must therefore be conceived as a living framework—one that continually assimilates longitudinal biomarker readouts, wearable-device metrics, and lifestyle variables to recalibrate therapeutic priorities in real time. This adaptive, systems-level perspective inevitably directs us to the next focal point: weaving pharmacologic agents, gene-targeted interventions, and lifestyle optimization into uniquely tailored therapeutic blueprints for each patient.

### 4.3. Integration of Multi-Modal Therapies: Pharmacological, Genetic, Lifestyle Interventions

Cutting-edge pharmacology will carry limited clinical weight unless it is braided together with gene-targeted tools and behaviorally anchored interventions. Network-based deep-learning frameworks already point the way: by training a multi-modal graph neural network on drug–gene–phenotype relationships, Balcı et al. uncovered repurposed compound pairs whose collective target map mirrors the multiplex pathology of PD rather than a single molecular choke-point [381]. Such in silico triage is beginning to inform nanocarrier platforms able to co-encapsulate kinase inhibitors, lysosomal chaperones, and anti-inflammatories in programmable ratios, thereby synchronizing pharmacokinetics and reducing “therapeutic drift” between agents [382]. Parallel advances in patient-derived induced pluripotent stem cells and CRISPR-based allele correction promise bespoke biologics that can be layered onto these smart-delivery systems, but risk–benefit modeling still lags behind technological exuberance [383]. To close this gap, adaptive trial designs must incorporate digital biomarkers that capture both molecular engagement and real-world function, transforming combination therapy from an empirical art into a data-driven science [384].

Equally crucial is the psychosocial fabric into which these molecular strategies are woven. Rhythmic-auditory cueing within group music-physiotherapy sessions can re-entrain gait circuitry while fostering social reward loops, all under the supervision of a single therapist [385]. Meta-analytic evidence confirms that aerobic exercise, resistance training, mindfulness practice, and speech therapy confer additive or even synergistic benefits across mobility, depression, and sleep architecture—outcomes that no pill, however sophisticated, achieves in isolation [281,385,386,387]. Integrated-care models staffed by movement-disorder specialists, physiotherapists, psychologists, and nutritionists not only improve motor scales but also trim emergency-department visits and caregiver burden, underscoring the economic imperative of multidisciplinarity [327]. Yet current reimbursement pathways still silo “medical” and “lifestyle” services, throttling access to comprehensive programs. Re-engineering healthcare policy to reward outcome-based bundles, while embedding wearable-derived metrics into routine follow-up, will be pivotal if we are to translate bench-born multimodal strategies into the lived experience of every person with PD. In short, the field must move from parallel experimentation to orchestrated choreography—aligning drug cocktails, gene editing, and rehabilitative regimens in a single, evolving score that plays to the rhythm of each individual’s disease trajectory.

## 5. Bridging Research Gaps: Strategic Recommendations

Bridging the persistent gaps in Parkinson’s research requires more than isolated technical innovation. First, studies must champion uncompromising methodological rigor and transparent reproducibility to ensure algorithms survive beyond their training sets. Equally critical is the adoption of unified clinical outcomes and biomarker criteria that permit immediate cross-trial comparability. These foundations only gain force when embedded in truly interdisciplinary, globally representative consortia that democratize data, training, and infrastructure. Finally, structured, bidirectional frameworks must accelerate the journey from bench discoveries to bedside interventions, shrinking the translational valley of death.

### 5.1. Enhancing Methodological Rigor and Reproducibility

Reproducibility remains the Achilles’ heel of PD research; progress stalls when algorithms flourish in silico yet collapse in an independent cohort. Multi-omic graph models that disclose their code, training splits, and external-validation sets—such as the open-access framework predicting prodromal PD across three international biobanks—illustrate a workable antidote to this malaise [333]. Ensemble pipelines built on stacking or hybrid architectures consistently outperform single-model efforts only when each layer is stress-tested with repeated cross-validation and bootstrapped confidence intervals [388,389]. Equally instructive is the finding that integrating heterogeneous SNP panels from multiple datasets, rather than cherry-picking the largest, markedly improves replication of biomarker signatures [296]. Together, these examples argue that methodological transparency, dataset integration, and adversarial validation must be codified as default—not aspirational—practices in PD data science.

Image-based biomarkers tell the same story. Seven-Tesla CEST of neurometabolic pools gains ten-fold statistical power once an optimized post-processing pipeline corrects for B0 drift and motion artefacts [390]. In radiomics, ComBat harmonization quells site- and scanner-induced variance, boosting both feature stability and classification accuracy across gray-level discretization schemes [391,392]. Hybrid machine-learning systems that combine radiomic signatures with clinical metrics identify robust PD subtypes only after passing independent statistical stress tests [393], while reproduction–replication workflows in longitudinal fMRI studies expose subtle trajectory predictors that would otherwise remain anecdotal [394,395]. Methodological rigor, then, is far more than a box-checking exercise; it is the scaffolding that allows candidate biomarkers to survive the leap from exploratory datasets to multicenter trials. Solidifying these practices naturally raises the next imperative: creating universally accepted outcome scales and biomarker thresholds so that findings forged in one laboratory can be immediately interpretable—and actionable—in another.

### 5.2. Standardizing Clinical Outcomes and Biomarker Criteria

Uniform outcome metrics and biomarker definitions are the linchpins of reliable cross-trial synthesis, yet they remain unevenly applied across PD studies. Even established clinical scales diverge in rater training, timing, and anchoring, complicating meta-analysis and masking subtle treatment effects. The latest evidence-based diagnostic guideline from the German Society of Neurology advocates a tiered framework that pairs core motor criteria with mandatory imaging or biofluid corroboration—an approach that could serve as a universal template if adopted beyond national borders [396]. Complementary position papers urge harmonization of prodromal criteria, the systematic incorporation of genetic red flags, and the calibration of imaging thresholds to scanner field strength [216]. Without such convergence, trial populations will continue to differ in invisible but outcome-critical ways.

Biomarker standardization is equally pressing. Longitudinal studies demonstrate that multiplex panels combining neurofilament light chain, APOE genotype, and clinical phenotype markedly outperform single-analyte measures for prognostic modeling and patient stratification [397]. Yet assay drift across laboratories still undermines replicability. Consensus protocols for CSF handling and assay quantitation, already drafted for α-syn species, must be extended to newer candidates such as L1CAM-positive exosomal cargoes [398,399,400]. Digital gait signatures and smartphone-derived motor diaries promise granular readouts of disease dynamics, but heterogeneity in sensor placement, sampling frequency, and analytic pipelines currently blunts their translational value [321,401]. Equally fragmented are the genetic and serological panels designed to predict cognitive decline; here, longitudinal validation and integration into unified assessment batteries remain conspicuous gaps [402]. In short, the field needs a Rosetta Stone that aligns clinical scales with fluid, genetic, and digital biomarkers under a single calibration charter. Crafting such a charter will demand more than technical expertise; it will require sustained dialogue among bioinformaticians, clinicians, biobank curators, and policymakers across diverse healthcare settings—a challenge that naturally ushers us toward a broader conversation on forging truly global and interdisciplinary research ecosystems.

### 5.3. Encouraging Interdisciplinary Collaboration and Global Representation

A truly transformative Parkinson’s research agenda cannot be confined to well-resourced academic islands; it must flow through an archipelago of disciplines, continents, and clinical realities [403,404]. The GP2 and MJFF Global Genetic projects supply a practical blueprint—cloud-based data portals, harmonized consent templates, and regional biobank hubs—that has already expanded sequencing efforts into Africa, Southeast Asia, and Latin America, regions long missing from the genetic narrative of PD [343,350,405]. Such infrastructures do more than diversify allele frequency maps; they seed capacity-building workshops, spawn junior-investigator exchanges, and weave local neurologists, geneticists, and data scientists into a unified knowledge loop [4,406]. Meanwhile, the World Health Organization’s recent roadmap underscores that equitable science requires parallel investment in care delivery, advocating cross-sector task forces that align public health, industry, and patient-advocacy agendas to close treatment gaps in low- and middle-income countries [407].

Interdisciplinarity must also penetrate day-to-day clinical practice. Network-care pilots in Germany reveal that structured communication channels linking neurologists, physiotherapists, speech therapists, and social workers slash referral delays and reduce hospital admissions, yet they hinge on designated “boundary spanners” who translate across professional jargon and electronic-record silos [328,335,336,408]. Embedding researchers within such networks accelerates bidirectional knowledge transfer: frontline therapists flag unmet needs, while investigators can prospectively enrol diverse patient cohorts into biomarker or digital-health studies without recruiting from scratch. Still, structural barriers remain—data-governance heterogeneity, uneven broadband access, and limited funding for transnational team science all threaten to slow momentum. Addressing these friction points will be vital if multicenter discoveries are to flow seamlessly toward clinical utility, a challenge that naturally directs our attention to the mechanisms required for efficient bench-to-bedside translation.

### 5.4. Developing Frameworks for Translating Bench Findings to Bedside Interventions

The translational “valley of death” in PD research reflects not a scarcity of discoveries but the lack of disciplined pipelines to escort them from Petri dish to prescription pad. Reviews tracking advances in etiology, microbiota–microglia crosstalk, and α-syn biology all stress that candidate therapies stumble when pre-clinical models fail to mirror the human milieu [409,410,411]. Age-, sex-, and behaviorally appropriate paradigms—aged rodents, female inclusion, impulse-control assays—sharpen pharmacodynamic read-outs and unmask toxicity before first-in-human dosing [412,413,414,415]. Bidirectional feedback is equally crucial: the stalled progress of clemizole in α-syn fibril models triggered back-translation into organoid systems, recalibrating dosage windows and engagement metrics prior to re-entry into the clinical queue [409,416,417]. Together these lessons argue that “bench to bedside” must be reconceived as an iterative, data-rich loop rather than a one-way conveyor belt.

**Figure 4:**
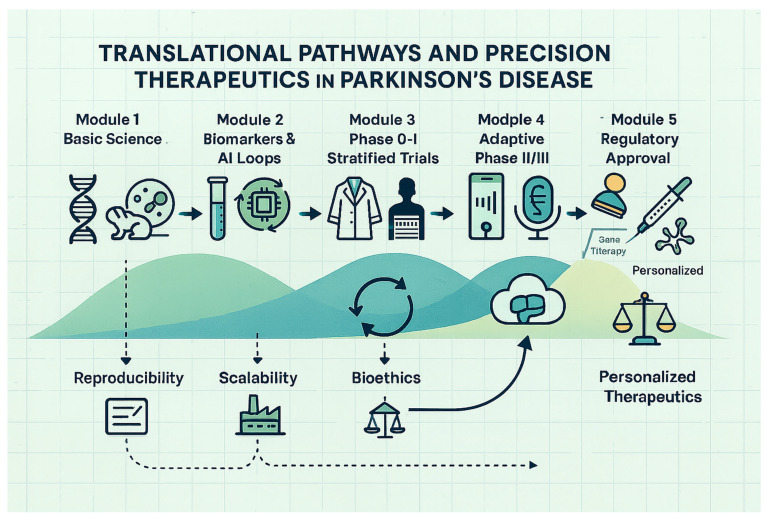
Translational pathways and precision therapeutics. Translational pathway from bench discoveries to precision therapeutics in Parkinson’s disease (PD). Sequential modules trace the journey: (1) basic science—omics, cell, and animal models; (2) biomarker discovery and target validation feeding forward into AI-driven drug-repurposing loops; (3) phase 0–I trials that stratify patients via molecular profiles; (4) adaptive phase II/III trials integrating digital gait, speech, and REM-metrics as endpoints; and (5) regulatory approval delivering individualized interventions (gene therapy, antisense, α-syn immunotherapy). Icons flag persistent hurdles—reproducibility, scalability, bioethical oversight—emphasizing the need for iterative feedback between preclinical, clinical, and real-world evidence streams to accelerate disease-modifying success. REM, rapid eye movement.

Multi-layered frameworks that fuse computational inference with experimental validation now promise to make that loop operational. CISL-PD’s counterfactual Dual GAN platform, for example, merges genomic, proteomic, and digital-phenotype streams to forecast intervention responses and nominate the most tractable clinical prototypes, substantially reducing the number of dead-end compounds entering costly toxicology screens [418]. Complementary blueprints embed circuit modeling, microbiome mapping, and neuroimaging surrogates within adaptive phase-II designs, allowing mechanistic biomarkers and clinical end-points to co-evolve in real time [419,420,421]. Human brain-based organoids provide a translational “middle kingdom,” bridging simplistic cell lines and heterogeneous patient cohorts while enabling parallel testing of dose, delivery, and off-target profiles [409]. When these assets feed into shared data lakes annotated with standardized metadata, they form a scalable scaffold adoptable by academic labs and industry alike—reducing redundancy, amplifying reproducibility, and accelerating the moment when mechanistic insight crystallizes into tangible therapeutic benefit (Figure 4).

## 6. Conclusions and Future Perspectives

Parkinson’s science stands at a decisive inflection point: dazzling molecular discoveries coexist with stubborn translational bottlenecks, and bridging that divide now defines the discipline’s grand challenge. Methodologically, reproducibility across laboratories, cross-ancestry genetics, and harmonized digital biomarkers remain under-built bridges; theoretically, the field still lacks a unifying systems model capable of weaving mitochondrial failure, α-syn misfolding, and neuroimmune crosstalk into one dynamic storyboard. Yet opportunity abounds. AI platforms that fuse multi-omic and wearable-sensor data, nanocarrier cocktails that co-deliver gene and small-molecule payloads, and patient-directed care networks already hint at a future in which interventions are not merely disease-modifying but person-specific and course-correcting in real time. Drawing on perspectives that span basic neuroscience, clinical neurology, computational biology, and rehabilitation sciences, the present author has critically appraised both mechanistic nuance and bedside pragmatism to chart actionable priorities. Researchers are urged to validate findings in diverse cohorts and aged, human-relevant models; clinicians to embed standardized outcome sets and patient-reported metrics in routine care; and policymakers to bankroll open-data infrastructures that democratize participation worldwide. Only through such concerted, interdisciplinary momentum is the promised era of transformative, individualized therapy for PD likely to shift from horizon to clinic room.

## Figures and Tables

**Table 1:** Genetic landscape of Parkinson’s disease (PD). Overview of key genetic contributors to PD, divided into Mendelian mutations and polygenic risk variants. Mendelian genes exhibit varying degrees of penetrance and clinical phenotype specificity, while genome-wide association studies (GWAS) highlight common risk alleles that converge on biological pathways. A final row summarizes current polygenic risk stratification tools. AUC, area under the curve; α-syn, α-synuclein; BST1, bone marrow stromal cell antigen 1 (CD157); GBA1, glucosylceramidase beta 1; GCH1, GTP cyclohydrolase 1; HLA-DRB5, major histocompatibility complex, class II, DR beta 5; LRRK2, leucine-rich repeat kinase 2; MAPT, microtubule-associated protein tau; MHC, major histocompatibility complex; PINK1, PTEN-induced kinase 1; PRKN, Parkin RBR E3 ubiquitin-protein ligase; PRS, polygenic risk score, SNCA, synuclein alpha gene; TMEM175, transmembrane protein 175.

Category	Gene/Locus	Penetrance/Effect Size	Notable Phenotype or Pathway
Mendelian Genes	SNCA	High (autosomal dominant)	Early-onset, rapid progression; α-syn aggregation
	LRRK2	Moderate to high (age-dependent)	Variable onset; kinase signaling, autophagy dysregulation
	PRKN	High (recessive)	Juvenile onset, slow progression; mitochondrial quality control
	PINK1	High (recessive)	Early-onset with dystonia; mitophagy dysfunction
	GBA1	Moderate (heterozygous), high (biallelic)	Cognitive decline risk; lysosomal storage pathway
Top GWAS Loci	MAPT (17q21)	OR ~1.3	Tau processing, microtubule stabilization
	BST1 (4p15)	OR ~1.2	Immune regulation and calcium signaling
	GCH1 (14q22)	OR ~1.1–1.3	Dopamine biosynthesis (tetrahydrobiopterin synthesis)
	TMEM175	OR ~1.1	Lysosomal function, linked to GBA1 network
	HLA-DRB5	OR ~1.2	Immune/inflammatory modulation, MHC class II region
Polygenic Risk Tools	–	Aggregated PRS (AUC ~0.65–0.70)	Integrate >80 loci; used in research stratification, biomarker enrichment

**Table 2:** Environmental and lifestyle exposures in Parkinson’s disease (PD). Summary of key environmental and lifestyle exposures linked to PD risk, including estimated relative risk (RR) ranges and hypothesized mechanistic pathways. The table distinguishes between toxicants with persistent neurotoxic effects and modifiable lifestyle factors with potential for risk reduction. α-syn, α-synuclein; BBB. blood–brain barrier; NO_2_, nitrogen dioxide; PM2.5, particulate matter ≤ 2.5 micrometers; TBI, traumatic brain injury; TCE, trichloroethylene.

Exposure Type	Example/Source	Relative Risk (RR)	Primary Mechanistic Link
Pesticides	Paraquat, rotenone	1.5–2.5	Mitochondrial complex-I inhibition, oxidative stress
Solvents	TCE, perchloroethylene	1.3–2.0	Dopaminergic neuron degeneration, α-syn aggregation
Metals	Manganese, lead	1.2–1.8	Oxidative damage, metal-induced neuroinflammation
Air Pollution	PM2.5, NO_2_	1.1–1.6	Microglial activation, systemic inflammation
Head Trauma	Repeated concussions, TBI	1.5–3.0	BBB disruption, tauopathy
Diet	High dairy, low antioxidants	0.8–1.3	Gut–brain axis, mitochondrial stress
Exercise	Moderate-to-vigorous activity	0.6–0.8 (protective)	Neurotrophic support, mitochondrial biogenesis

Note: Pesticides, solvents, metals, air pollution, and head trauma typically represent irreversible exposures with cumulative neurotoxic effects. In contrast, diet and exercise are modifiable lifestyle factors that offer opportunities for risk mitigation and neuroprotection.

**Table 3:** Neuroinflammation, oxidative stress, and candidate modulators in Parkinson’s disease (PD). Overview of major neuroinflammatory and oxidative stress pathways implicated in PD, paired with selected therapeutic agents that have reached at least Phase I clinical evaluation. The table highlights emerging strategies to modulate innate immune activation, redox imbalance, and mitochondrial dysfunction. ARE, antioxidant response element; ApTOLL, aptamer targeting TLR4; GSK-3β, glycogen synthase kinase 3 beta; IL, interleukin; NADPH, nicotinamide adenine dinucleotide phosphate; NLRP3; NACHT, LRR and PYD domains-containing protein 3; NOX2, NADPH oxidase 2; NRF2, nuclear factor erythroid 2-related factor 2; ROS, reactive oxygen species; TLR4, Toll-like receptor 4.

Pathway/Target	Mechanistic Role	Therapeutic Lead (≥Phase I)	Mechanism of Modulation
TLR4 (Toll-like receptor 4)	Innate immune activation, microglial priming	ApTOLL (TLR4 antagonist)	Blocks pro-inflammatory signaling cascade
NLRP3 inflammasome	IL-1β/IL-18 maturation, pyroptosis	Inzomelid (Inflazome/Roche)	Selective NLRP3 inhibition
NRF2	Antioxidant transcriptional response	Dimethyl fumarate, PB125	Activates NRF2-ARE pathway
Mitochondrial Complex I	Site of rotenone toxicity, ROS overproduction	UBIAD1 analogs, IACS-010759	Stabilize complex I/enhance respiratory flux
GSK-3β	Crosstalk between inflammation and oxidative stress	Tideglusib, LY2090314	Inhibits GSK-3β to restore redox and immune balance
NOX2 (NADPH oxidase 2)	ROS generation in activated microglia	GSK2795039 (NOX2 inhibitor)	Attenuates microglia-derived oxidative burst

**Table 4:** Core research gaps and consequences in Parkinson’s disease (PD). Key methodological and translational gaps that currently distort the PD evidence base. Each gap is paired with its downstream effect on interpretation or replication and a concrete corrective action as outlined in the review. iPSC, induced pluripotent stem cell.

Core Gap	How It Skews Evidence	Concrete Fix Suggested in Review
Diagnostic variability	Inflates cohort heterogeneity; undercuts power in early-phase trials	Apply multidimensional stratification (clinical + molecular)
Animal–human mismatch	Overpredicts efficacy; fails to capture complex non-motor pathology	Employ human iPSC-derived neurons/organoids and aged animal models to replicate human, age-dependent Parkinson’s pathology
Biomarker drift	Leads to irreproducible panels; fails external validation	Use longitudinal anchoring and cross-platform harmonization
Phase II–III collapse	Promising leads fail at scale; endpoint misalignment	Integrate target engagement biomarkers + adaptive designs
Underrepresentation	Skews generalizability; neglects frailty and late-life phenotypes	Mandate inclusive recruitment across age, ethnicity, frailty
Compartmentalized datasets	Blocks integration across imaging, omics, clinical tools	Build multimodal, federated data architectures

**Table 5:** Current validation phases of leading Parkinson’s disease (PD) biomarkers. This summary groups the most widely investigated molecular, imaging, and digital biomarkers by modality and read-out, indicating whether each is still in preclinical testing, early human trials, or already clinically qualified for routine use.

Biomarker/Tool	Modality	Primary Target/Read-out	Current Validation Phase
CSF α-syn RT-QuIC	Biofluid (CSF)	Seed-competent α-syn aggregates	Phase II (multicenter observational cohorts)
Plasma p-α-syn	Biofluid (blood)	Phosphorylated α-syn species	Phase I (assay standardization)
Plasma (NfL)	Biofluid (blood/CSF)	Axonal integrity marker	Clinically qualified for prognostics in ALS; Phase II for PD
DAT-SPECT	Molecular Imaging	Striatal dopamine-transporter binding	Clinically qualified (diagnostic aid)
Neuromelanin-sensitive MRI	Advanced MRI	Nigral neuromelanin loss	Phase I/II (single-site reproducibility)
α-syn PET Tracers	Molecular Imaging	Fibrillar/oligomeric α-syn in vivo	Preclinical → early Phase I (first-in-human safety)
Wearable-derived Gait Metrics	Digital/Sensor	Step amplitude, stride dynamics	Phase I/II (analytical validity, small cohorts)
Speech-analytics (smartphone)	Digital/AI	Articulatory rate, pitch variability	Phase I (algorithm development)

AI, artificial intelligence; ALS, amyotrophic lateral sclerosis; CSF, cerebrospinal fluid; α-syn, alpha-synuclein; MRI, magnetic resonance imaging; NfL, neurofilament light chain; PET, positron emission tomography.

**Table 6:** Diversity and representation in Parkinson’s disease (PD) research. Summary of current representation gaps and inclusion efforts across key domains of PD research. The table compares non-European ancestry participation in genetic, biomarker, and clinical trial datasets, and highlights major initiatives actively addressing global equity and inclusion. CSF, cerebrospinal fluid; FIRE-UP PD, Fostering Inclusivity in Research Engagement for Underrepresented Populations in Parkinson’s Disease; GP2, Global Parkinson’s Genetics Program; GWAS, genome-wide association studies; MJFF, Michael J. Fox Foundation.

Research Domain	Current Non-European Participation	Active Inclusion Initiatives
Genetic Studies	<15% globally; <5% in GWAS meta-analyses	GP2—expanding genomic data from Africa, Asia, Latin America
Biomarker Cohorts	<10% in most CSF/imaging studies	MJFF Global PD Initiative—building diverse biosample banks and imaging pipelines
Clinical Trials	Typically <8% non-European enrolment	FIRE-UP PD—focused on equitable recruitment, community engagement, and outcome relevance

## Data Availability

Not applicable.

## Abbreviations

The following abbreviations are used in this manuscript:

18F-FDG	18-fluorodeoxyglucose
AI	artificial intelligence
α-syn	alpha-synuclein
COMT	catechol-O-methyltransferase
CSF	cerebrospinal fluid
DAT	dopamine transporter
FDG	fluorodeoxyglucose
GBA1	glucocerebrosidase 1
GIP	glucose-dependent insulinotropic polypeptide
GLP-1	glucagon-like peptide-1
GP2	Global Parkinson’s Genetics Program
GWAS	genome-wide association studies
IoT	Internet-of-Things
LRRK2	leucine-rich repeat kinase 2
MDS	Movement Disorder Society
MDS-UPDRS	Movement Disorder Society–Unified Parkinson’s Disease Rating Scale
MRI	magnetic resonance imaging
MPTP	1-Methyl-4-phenyl-1,2,3,6-tetrahydropyridine
NLRP3	NACHT, LRR and PYD domains-containing protein 3
NRF2	nuclear factor erythroid 2-related factor 2
PD	Parkinson’s disease
PET	positron emission tomography
PINK1	PTEN-induced kinase 1
PRKN	Parkin RBR E3 ubiquitin-protein ligase
RBD	REM-sleep behavior disorder
REM	rapid eye movement
SNCA	synuclein alpha gene
SNP	single-nucleotide polymorphism
